# Genetic and Phytochemical Characterization of Lettuce Flavonoid Biosynthesis Mutants

**DOI:** 10.1038/s41598-019-39287-y

**Published:** 2019-03-01

**Authors:** Csanad Gurdon, Alexander Poulev, Isabel Armas, Shukhratdzhon Satorov, Meg Tsai, Ilya Raskin

**Affiliations:** Department of Plant Biology, Rutgers University, 59 Dudley Road, New Brunswick, NJ 08901-8520 USA

## Abstract

We previously developed red lettuce (*Lactuca sativa* L.) cultivars with high flavonoid and phenolic acid content and demonstrated their anti-diabetic effect. Here we report on developing three fertile and true-breeding lettuce lines enriched with flavonoids with reported beneficial health effects. These lines were identified in a segregating population of EMS-mutagenized red lettuce and characterized biochemically and genetically. Change in red coloration was used as a visual indicator of a mutation in a flavonoid pathway gene, leading to accumulation of flavonoid precursors of red anthocyanins. Pink-green kaempferol overproducing *kfoA* and *kfoB* mutants accumulated kaempferol to 0.6–1% of their dry weight, higher than in any vegetable reported. The yellow-green naringenin chalcone overproducing mutant (*nco*) accumulated naringenin chalcone, not previously reported in lettuce, to 1% dry weight, a level only observed in tomato peel. *Kfo* plants carried a mutation in the *FLAVONOID-3′ HYDROXYLASE* (*F3′H*) gene, *nco* in *CHALCONE ISOMERASE* (*CHI*). This work demonstrates how non-GMO approaches can transform a common crop plant into a functional food with possible health benefits.

## Introduction

Fruits and vegetables are good dietary sources of phenolics, ubiquitous phytochemicals that include flavonoids and phenolic acids^1^. Epidemiological studies suggested that diets high in fruits and vegetables confer beneficial effects on chronic metabolic and cardiovascular diseases^2–4^. With some exceptions^5^, these benefits were confirmed by meta-analyses of cohort studies^6–8^. However, the average dietary flavonoid consumption in Europe and the US may be too low to confer health effects^9,10^.

Lettuce (*Lactuca sativa*, family Asteraceae) is a crop domesticated more than ten thousand years ago^11^. It is the third most commonly consumed vegetable in the US after potato and tomato, with US per capita consumption estimated at 11.7 kg/year^12^, and is considered a good source of fiber, iron, folic acid and vitamin C^13^. Common lettuce phenolics are caffeic acid derivatives, predominantly chicoric, chlorogenic, caffeoyltartaric and caffeoylmalic acids; and flavonol glycosides, predominantly quercetin 3-O-malonylglucoside, quercetin 3-O-glucoside and quercetin 3-O-glucuronide^14,15^. In addition, red varieties contain the anthocyanin cyanidin 3-O-malonylglucoside^14,15^. Flavonoid and total phenolics levels vary widely between lettuce types: crisphead varieties, commonly consumed in the US, have low levels of phenolics, while red leaf and red oak lettuces have the highest levels^13,15^. Thus, there is potential to develop cultivars with enhanced nutritional or functional value (see e.g.^16^). Earlier we have developed three Rutgers Scarlet Lettuce (RSL) lines from existing red cultivars using tissue culture selection for deep purple color, an indicator of high anthocyanin content^17^. In addition to the anthocyanin cyanidin 3-O-malonylglucoside, RSL lines accumulated high levels of common phenolics reported in lettuce^14,15,18^, such as quercetin glycosides and chlorogenic acids, resulting in a total phenolic content of >9% dry leaf weight, the highest reported in the literature^17^. RSL leaf and extract showed *in vivo* anti-diabetic effect in a mouse model of type 2 diabetes^17,18^. Specifically, daily oral administration of RSL extract to obese C57BL/6 mice kept on High Fat Diet (HFD) for 28 days resulted in improved oral glucose tolerance and decreased liver lipid levels compared to control^17,18^. Thirteen-week diet supplementation with RSL powder resulted in improved glucose tolerance in obese C57BL/6 mice on HFD, even though other measured physiological parameters did not change^19^. In another study, 4-week supplementation with red leaf lettuce powder resulted in decreased levels of total blood cholesterol and triglycerides in HFD-fed mice^20^.

The aim of this study was to develop fertile and true-breeding lettuce varieties enriched in specific flavonoids beneficial for human health, such as kaempferol and naringenin chalcone, present only in small or undetectable amounts in wild type lettuce, as even structurally similar flavonoids can produce markedly different health effects^21,22^.

**Figure 1:**
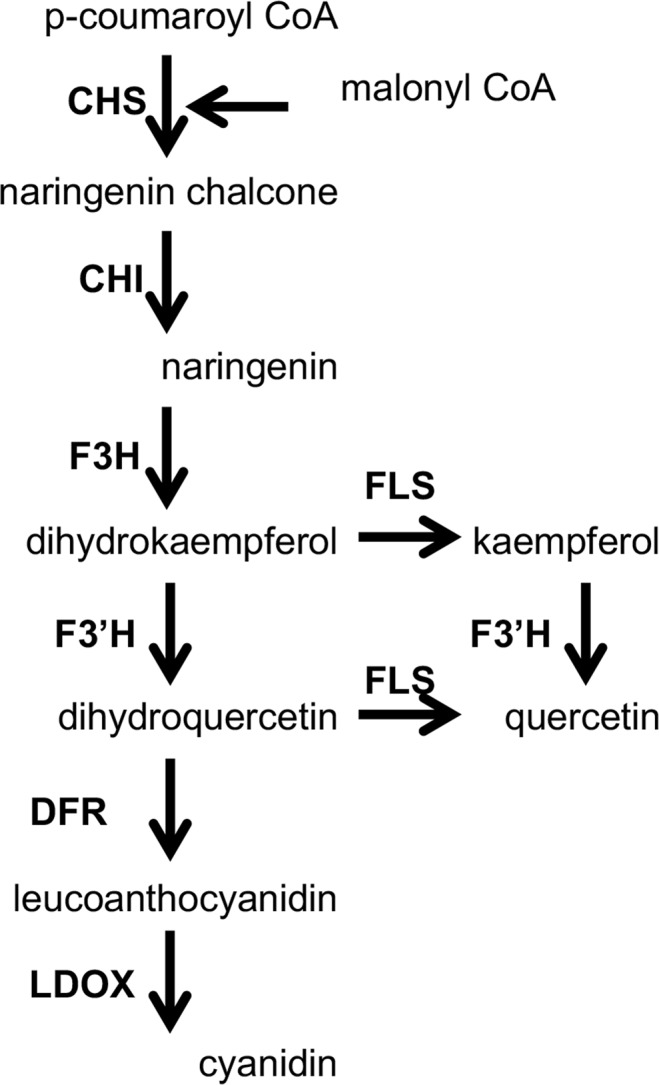
Enzymes of the flavonoid biosynthesis pathway in *A*. *thaliana* adapted from^25^. All enzymes but FLS are coded by a single gene in *A*. *thaliana*. Genes coding for the enzymes responsible for glycosylation, acylation, and intracellular transport of flavonoids are not shown. Abbreviations: CHS, CHALCONE SYNTHASE; CHI, CHALCONE ISOMERASE; F3H, FLAVANONE 3-HYDROXYLASE; F3′H, FLAVONOID 3′-HYDROXYLASE; FLS, FLAVANOL SYNTHASE; DFR, DIHYDROFLAVONOL REDUCTASE; LDOX, LEUCOANTHOCYANIN DEHYDROGENASE.

As flavonoid biosynthesis genes have been characterized in *Arabidopsis thaliana* (Fig. 1) for review, see^23–25^, we designed primers based on lettuce homologs of Arabidopsis genes, and identified mutations in lettuce *CHALCONE ISOMERASE (CHI)* and *FLAVONOID-3′ HYDROXYLASE* (*F3′H*) genes responsible for the novel phenotypes. These mutations increased the levels of targeted flavonoids to levels higher than in any other vegetable. Recent sequencing of the lettuce genome^26^ and identification of loci associated with the flavonoid biosynthesis pathway in lettuce^11^ allowed us to compare the *CHI* and *F3′H* sequences we determined to those putatively identified by Zhang *et al*.^11^.

## Results

### Isolation of flavonoid biosynthesis mutants

An ethyl methanesulfonate (EMS)-mutagenized *cv*. Firecracker red leaf lettuce segregating population derived from seeds of self-pollinated mutagenized plants was screened for anthocyanin (cyanidin 3-O-malonylglucoside) loss manifested by changes in color. 1522 mutagenized (M1) plants were grown from seed mutagenized by 0.10 or 0.15% EMS, selfed, and the mature dry inflorescences collected to obtain the M2 segregating population. 136 M1 lines were sterile. Seed from the remaining 1386 M1 lines were planted (12 seeds per plant, if available) in growth chambers equipped with cool fluorescent lights emitting high levels of both photosynthetically active radiation and ultraviolet (UV); lighting conditions known to induce strong anthocyanin accumulation, and, thus, red color. Forty-three lines harboring color variants were identified visually. Methanolic extracts of the twenty most prominent color mutants were biochemically profiled using an Ultra Performance Liquid Chromatography - Tandem Mass Spectrometer (UPLC-MS/MS) system. Three mutants were selected for further studies.

**Figure 2:**
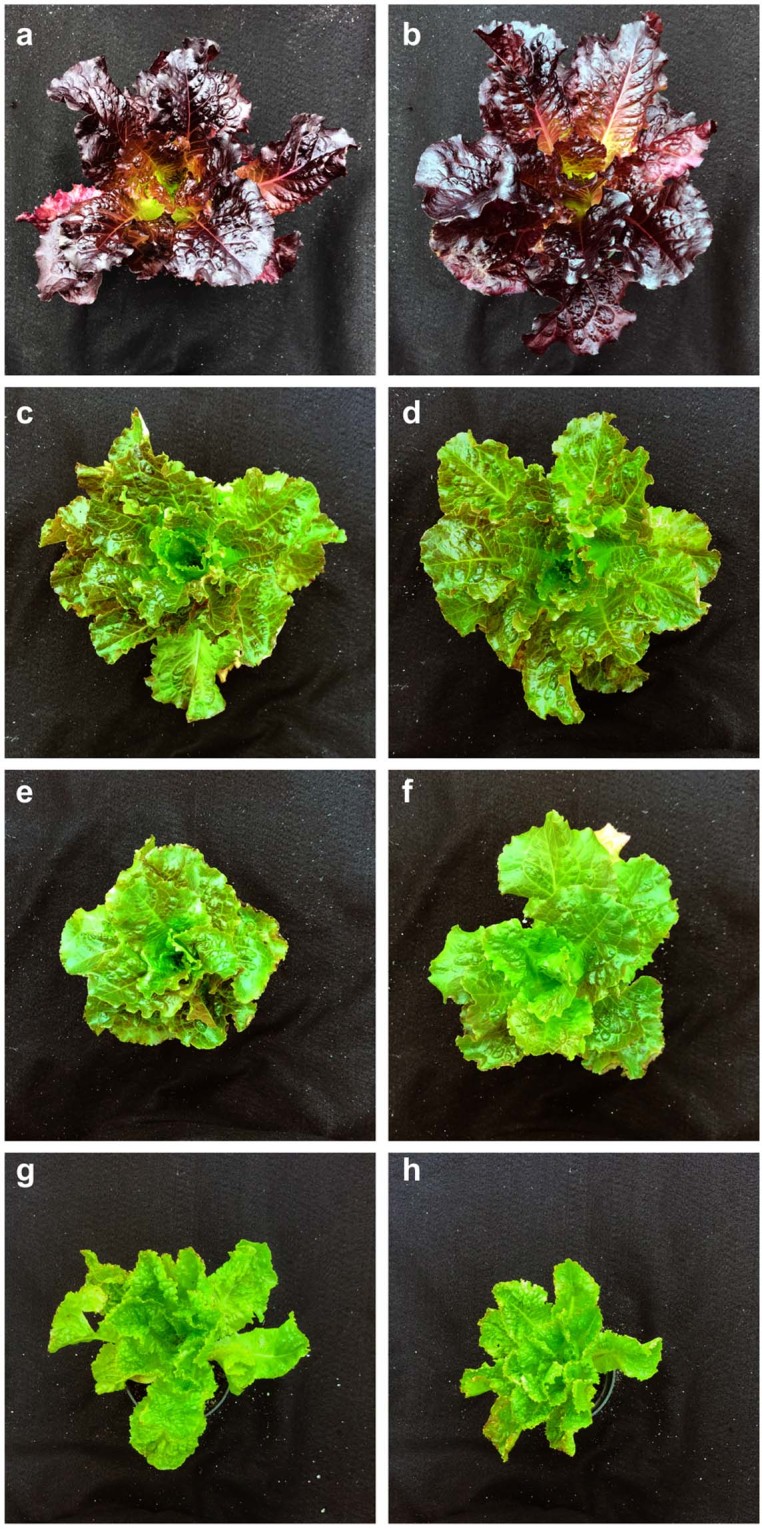
Representative 15-week old *cv*. Firecracker (wild type) and flavonoid biosynthesis mutants, grown under cool fluorescent lights. Two representative plants per line are shown photographed to scale. (**a**,**b**) *cv*. Firecracker; (**c**,**d**), *kfoA* (**e**,**f**); *kfoB*; (**g**,**h**), *nco*.

Pink-green kaempferol overproducer *kfoA* had high levels of kaempferol glycosides (mostly kaempferol 3-O-malonylglucoside, low amounts of kaempferol 3-O-glucoside and kaempferol 3-O-glucuronide) but lacked quercetin or cyanidin derivatives. Another kaempferol overproducer, *kfoB*, accumulated the same kaempferol derivatives as *kfoA*, and had low but detectable cyanidin and quercetin glycoside content. The yellow-green naringenin chalcone overproducer *nco* line had high levels of glycosylated compounds (hexosides and malonylhexoside) (Supplementary Fig. S1g,h,i), with a shared aglycone ion of m/z 273 [M + H] (Supplementary Fig. S1m,n), which corresponds to the isomers naringenin chalcone (Supplementary Fig. S1l) and naringenin (Supplementary Fig. S1j). However, naringenin and naringenin chalcone have characteristically different UV spectra, naringenin chalcone having its absorption maximum at 365 nm (Supplementary Fig. S1q), and naringenin at 289 nm (Supplementary Fig. S1o). Additionally, the UV absorbance spectra of naringenin glycosides and naringenin chalcone glycosides are similar to the spectra of their aglycones^27^ and Supplementary Fig. S1p. Both glycosides in *nco* lettuce had the characteristic UV absorbance spectra of naringenin chalcone (Supplementary Fig. S1r,s). Based on these data and on genetic data below, we concluded that *nco* lettuce accumulated naringenin chalcone glycosides. *Nco* lacked detectable kaempferol or cyanidin derivatives and had greatly reduced quercetin level compared to *cv*. Firecracker. Supplementary Fig. S2 shows major peaks of *cv*. Firecracker, *kfo* and *nco* extract chromatograms. Accumulation of high levels of kaempferol or naringenin chalcone is a novel trait in lettuce^28^, therefore, *kfoA*, *kfoB* and *nco* were further characterized. Figure 2 shows representative photos of 15-week old *cv*. Firecracker, *kfoA*, *kfoB* and *nco* plants grown under UV-emitting, cool fluorescent lights. Under these conditions, Firecracker plants were deep red (Fig. 2a,b), *kfoA* (Fig. 2c,d) and *kfoB* (Fig. 2e,f) were pink-green, and *nco* (Fig. 2g,h) were yellow-green color. All mutants grew slower than wild type *cv*. Firecracker plants under fluorescent lights (UV light intensity 0.4 ± 0.1 mol/m^2^d), a trait previously described in *A*. *thaliana* flavonoid biosynthesis mutants^29,30^.

### *KfoA* and *nco* accumulate high amounts of flavonoid compounds missing from parental line *cv*. Firecracker

*KfoA*, *kfoB* and *nco* mutants and wild type *cv*. Firecracker were grown under identical conditions illuminated by cool fluorescent lights and subjected to further UPLC-MS/MS analysis. Leaves were harvested from 18-week old plants, lyophilized, mixed with HCl-acidified methanol, and subjected to acid hydrolysis, based on the method of Hertog *et al*.^31^. This treatment results in the removal of glycosylation from all flavonoids and chalcones, allowing for the quantification of aglycones, or, in case of *nco*, their derivatives using UPLC-MS/MS (Table 1). Supplementary Fig. S3 shows representative chromatograms of *cv*. Firecracker, *kfoA*, *kfoB* and *nco* acid hydrolyzed extracts.

**Table 1:** Flavonoid aglycones in 18-week old red *cv*. Firecracker, *kfoA*, *kfoB* and *nco* lettuce grown under cool fluorescent lights. Acid hydrolysis was used to convert compounds to aglycones.

Lines	Cyanidin	Quercetin	Kaempferol	Naringenin	Pelargonidin	Total polyphenol
*cv*. Firecracker	5.3 ± 2.5 (57.49 ± 26.41)	26.0 ± 2.5 (281.53 ± 47.83)	<0.02 BQ	<0.04 BQ	<0.2 BQ	45.00 ± 8.51
*kfoA*	<0.02 BQ (<0.18)	<0.02 BQ (<0.18)	10.9 ± 2.9 (102.62 ± 32.47)	<0.04 BQ	0.30 ± 0.1 (3.38 ± 2.18)	23.33 ± 7.45
*kfoB*	0.14 ± 0.01 (1.80 ± 0.29)	0.3 ± 0.2 (3.79 ± 2.15)	6.4 ± 1.5 (83.41 ± 25.31)	<0.04 BQ	0.58 ± 0.2 (7.42 ± 2.41)	44.87 ± 3.75
*nco*	<0.02 BQ (<0.18)	0.6 ± 0.2 (5.59 ± 1.34)	<0.02 BQ (<0.18)	10.4 ± 2.5 (92.73 ± 22.81)	<0.02 BQ	36.29 ± 8.57

Mean mg compound/ g dry leaf weight, and, in parenthesis, as mg compound/ 100 g fresh leaf weight ± standard deviation is shown for *cv*. Firecracker (n = 6), *kfoA*, *kfoB* and *nco* (n = 10). Pelargonidin was quantified in cyanidin equivalents. Total polyphenol content is calculated as gallic acid (GA) equivalent in mg GA/ g dry leaf weight. BQ, below quantification limits. Naringenin chalcone glycosides were converted to naringenin during acid hydrolysis.

The anthocyanin cyanidin and the flavonol quercetin were detected in *cv*. Firecracker extracts, as expected in red leaf lettuce^14,15,17^. Additionally, low levels of pelargonidin were observed (Table 1). In *kfoA* plants cyanidin and quercetin were not detectable. Instead, accumulation of the flavonol kaempferol and the anthocyanidin pelargonidin was observed. While kaempferol has been reported in lettuce^28,32–34^, *kfoA* plants accumulated >10 mg kaempferol/ g dry weight, or ~103 mg kaempferol/100 g fresh weight, two orders of magnitude higher than previously reported. Additionally, *kfoA* and *kfoB* plants contained more pelargonidin (0.33 and 0.60 mg pelargonidin/g dry weight), the predominant anthocyanin in strawberries^35^, than *cv*. Firecracker (<0.2 mg pelargonidin/g dry weight). *KfoB* plants accumulated >6 mg kaempferol/g dry weight, lower than *kfoA*. However, they accumulated more pelargonidin than *kfoA*, and contained quantifiable cyanidin and quercetin. To our best knowledge, this is the first report on the accumulation of pelargonidin in lettuce leaves.

*Nco* acid hydrolyzed extracts lacked cyanidin, kaempferol or pelargonidin, but contained >10 mg naringenin/g dry weight, and, on average, 0.6 mg quercetin/g dry weight. As naringenin chalcone glycosides, but not naringenin glycosides were observed in non-hydrolyzed *nco* extracts (see previous section), we tested the effect of acid hydrolysis on pure naringenin chalcone and observed full conversion to naringenin. Therefore, the levels of naringenin in hydrolyzed extracts of *nco* correspond to the levels of naringenin chalcone glycosides in the plant. Small amounts of quercetin observed in *nco* were also likely derived from naringenin formed spontaneously *in planta* from naringenin chalcone, as naringenin chalcone can spontaneously isomerize by C ring closure to naringenin^36^. To our best knowledge, naringenin chalcone has not been described in lettuce before.

Total polyphenol levels were measured in ten plants per line, using a modified Folin-Ciocalteu assay^17^. Wild type *cv*. Firecracker and *kfoB* both had 45 mg gallic acid equivalent/g dry weight. *KfoA* and *nco* plants had somewhat lower total polyphenol levels: 23 and 36 mg gallic acid equivalent/g dry weight, respectively (Table 1).

### *Nco* is a *chalcone isomerase* mutant

The *nco* flavonoid profile (Table 1) resembled *A*. *thaliana tt5* null mutants, which have nonfunctional CHALCONE ISOMERASE (CHI), an enzyme that converts naringenin chalcone to naringenin^29,37^. Therefore, primers designed based on lettuce Expressed Sequence Tags (ESTs) homologous to the *A*. *thaliana CHI* gene (TAIR AT3G55120) were used to amplify the full coding sequence (CDS) of the putative lettuce *CHI* from cDNA in *cv*. Firecracker and *nco*. The wild type *cv*. Firecracker *CHI* (*CHI*+, NCBI MG981123) was predicted to code for a 235-amino acid protein, and the CDS was identical to XM_023891334, a predicted CHALCONE ISOMERASE from green crisphead lettuce *cv*. Salinas. Additionally, it was identical to LG9_805610, identified as the only CHI expressed (of two putative CHI genes) in the lettuce genome^11^. *Nco* plants were homozygous for an allele (*chi*1, NCBI MG981124) that harbors a premature stop codon caused by a G to A mutation in codon 120, truncating the CHI enzyme. The CHI1 truncated protein lacks two conserved residues of the naringenin binding cleft, as well as a residue of the active site hydrogen bond network^38^; therefore, it is expected to be nonfunctional.

Of the M2 population, one *nco* mutant and 4 wild type siblings were genotyped. The mutant was homozygous for the *chi1* allele, whereas wild type plants were heterozygous or homozygous for *CHI*+ allele. The M2 mutant and its wild type red siblings were selfed, and segregation ratios in M3 individuals were observed. In addition, selfed seed from two M3 mutants were planted. (Table 2; Supplementary Table S1 for segregation ratios of individual parents). Homozygous *chi1* mutants always produced yellow-green offspring, heterozygotes produced yellow-green and red offspring, and homozygous *CHI*+ plants always produced red offspring, indicating that the mutant allele is recessive and responsible for the observed phenotype.

**Table 2:** Phenotype segregation ratios in *kfoA*, *kfoB* and *nco* lines. Summary of all lines is shown; segregation data for individual lines is shown in Supplementary Table S1.

Line	Parent genotype	Number of mutant offspring	Number of wild type offspring	Total number of offspring	Percentage of mutants
*kfoA*	+/M	32	53	85	37.6%
	+/+	n/a	n/a	n/a	n/a
	M/M	385	0	385	100.0%
*kfoB*	+/M	14	46	60	23.3%
	+/+	0	38	38	0.0%
	M/M	67	0	67	100.0%
*nco*	+/M	18	46	64	28.1%
	+/+	0	27	27	0.0%
	M/M	112	0	112	100.0%

**Figure 3:**
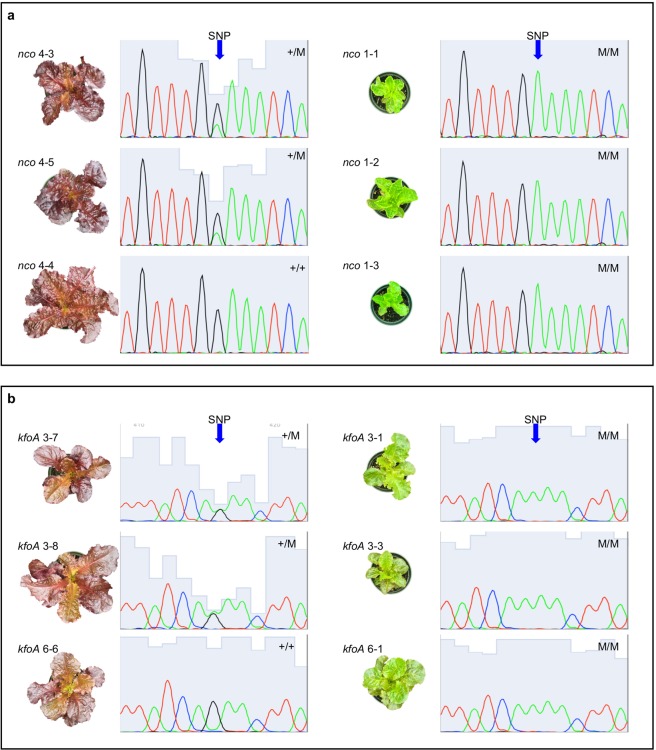
Phenotype of *kfo* and *nco* plants is determined by *F3′H* and *CHI* genotypes. (**a**) Representative photos, to scale, and genotyping chromatograms of wild type and yellow-green *nco* mutants. *CHI* homozygous recessive mutant genotype results in mutant yellow-green phenotype. Heterozygous and homozygous wild type genotypes result in wild type red phenotype. (**b**) Representative photos, to scale, and genotyping chromatograms of wild type and pink-green mutant *kfoA* line plants. *F3′H* splice site homozygous mutant genotype results in mutant pink-green phenotype. Heterozygous and homozygous wild type genotypes result in wild type red phenotype. Genotypes: +/+, homozygous wild type; +/M, heterozygous mutant; M/M, homozygous mutant. Note single peak in homozygotes, and double peak in heterozygotes at SNP site. Fifteen-week-old plants are shown, grown under cool fluorescent lights in identical conditions.

We then genotyped 5 mutants and 14 wild-type siblings from self-pollinated offspring of *nco* M2 plants (M3 generation) and found that only yellow-green mutants were homozygous for *chi1* (Fig. 3a). Additionally, we amplified the full CDS of the putative lettuce *F*3*H* gene in *cv*. Firecracker and *nco* from cDNA using primers designed based on lettuce ESTs homologous to the *A*. *thaliana FLAVANONE-3-HYDROXYLASE* (*F3H)* gene (TAIR AT3G51240). F3H converts naringenin to dihydrokaempferol (Fig. 1), and, in *A*. *thaliana*, *f3h* mutants accumulate a mix of chalcones, flavonols and anthocyanins^39^. As in Arabidopsis, *F3H* in lettuce is a single-copy gene^11^. The *F3H* coding sequence of *nco* was found to be identical to that of *cv*. Firecracker. Our data suggest that though two putative *CHI* copies exist in the lettuce genome^11^, losing both functional copies of the LG9_805610 gene leads to low anthocyanin (yellow-green) phenotype, and that *nco* is a *chi* mutant.

### *KfoA* and *kfoB* are *flavonoid 3′-hydroxylase* mutants

The *kfoA* flavonoid profile (Table 1) resembled *Arabidopsis thaliana tt7* null mutants, which have a nonfunctional FLAVONOID-3′ HYDROXYLASE (F3′H) enzyme^40,41^. Therefore, primers designed based on lettuce ESTs homologous to the *A*. *thaliana F3′H* gene (TAIR AT5G07990) were used to amplify the full CDS of the putative lettuce *F3′H* in *cv*. Firecracker, *kfoA* and *kfoB*. The wild type *cv*. Firecracker *F3′H* (*F3′H*+, NCBI MG981125) was a gene containing three exons, predicted to code for a 512-amino acid protein, and was identical to XM_023887166, a predicted FLAVONOID 3′-MONOOXYGENASE-LIKE gene from green crisphead lettuce *cv*. Salinas. Additionally, it was identical to LG5_471950, one of five putative *F3′H* genes in the lettuce genome identified by Zhang *et al*.^11^.

*KfoA* plants were homozygous for an allele (*f3′h1*, NCBI MG981126) harboring a G to A mutation in the splice acceptor site of intron 2, while *kfoB* plants were homozygous for an allele (*f3′h2*, NCBI MG981127) harboring a premature stop codon caused by a C to T mutation in codon 233. Translated *kfo* F3′H proteins harbor the CR1 active site (amino acids 171–186) responsible for the hydroxylating activity, but lack three substrate recognition sites as well as the EXXR motif necessary for core stabilization^42^, thus, it is expected that both *kfoA* and *kfoB* mutant F3′H proteins are nonfunctional. Of the M2 population, one *kfoA* mutant and seven wild type siblings were genotyped, as well as one *kfoB* and five wild type siblings. Mutants were homozygous for *f3′h1* or *f3′h2*, while wild type plants were heterozygous or homozygous for the wild type *F3′H*+. Mutant plants and wild type red siblings were selfed, and segregation ratios in M3 and M4 individuals were observed (Table 2; Supplementary Table S1 for segregation ratios of individual parents). Homozygous *f3′h* mutants always produced pink-green offspring, heterozygotes produced pink-green and red offspring, and homozygous wild type plants always produced red offspring, indicating that the mutant alleles are recessive and responsible for the observed phenotype. Genotyping 15 mutants and 5 wild-type siblings from self-pollinated offspring of *kfoA* M2 plants (M3 generation), we found that only pink-green mutants were homozygous for *f3′h1* (Fig. 3b). Our data suggest that *kfoA* and *kfoB* are mutants for one of the five *F3′H* gene copies in lettuce (LG5_471950 in^11^), and that this gene is predominantly responsible for the synthesis of dihydroquercetin, a precursor in anthocyanin biosynthesis in wild type *cv*. Firecracker.

## Discussion

*Nco* lettuce plants have a characteristic yellow-green leaf color due to the accumulation of yellow-colored naringenin chalcone glycosides and the lack of red anthocyanins. Naringenin chalcone has not been reported in lettuce before. Tomato (*Solanum lycopersicum*) skin is the best-known food source of this compound, where it accumulates up to 1% dry weight^43^, a level similar to that of *nco* lettuce. Naringenin chalcone is anti-inflammatory, anti-allergic (e.g.^44,45^) and anti-obesity^46^ *in vitro* and *in vivo*, and was found to improve symptoms of perennial allergic rhinitis in a clinical trial^47^. Therefore, *nco* lettuce could be a useful dietary source for naringenin chalcone, with one US leaf lettuce serving of 85 g containing ~79 mg of the compound.

The color phenotype in *nco* lettuce is caused by a nonsense mutation in the *CHI* gene. To our knowledge, *nco* is the first *chi* mutant in lettuce. While CHI is ubiquitous in higher plants, *chi* mutants have been characterized from just a handful of species, with individual flavonoids from these mutants not quantified. *Chi* mutants have been described in ornamental flowers such as *Petunia hybrida*^48^, *Callistephus chinensis*^49^ and *Dianthus caryophillus*^50^, in crops such as barley (*Hordeum vulgare*)^51,52^, rice (*Oryza sativa*)^53^ and onion (*Allium cepa*)^54^ and in*A*. *thaliana*^29,37,55^. In all species, the *chi* mutant phenotype results in yellowish tissues: hull and internodes in rice^53^, bulb color in onion^54^, petals in *C*. *chinensis*^49^ and *D*. *caryophillus*^50^, seed coat in *A*. *thaliana*^56^ and pollen in *P*. *hybrida*^48^. *P*. *hybrida chi* mutants accumulate naringenin chalcone as aglycone^48^, while *C*. *chinensis*^49^, *D*. *caryophillus*^50^ and barley (*Hordeum vulgare*)^51^ *chi* mutants accumulate naringenin chalcone 2′-glucoside (isosalipurposide). Detailed metabolome analysis of *A*. *thaliana chi* mutants revealed the presence of multiple naringenin chalcone glycosides^37^, while in *chi* onion^54^ and rice^53^ the compound responsible for the yellowish or golden color was not identified. We found that *nco* lettuces accumulate naringenin chalcone hexoside and malonylhexoside, but not the aglycone, similarly to most *chi* mutants.

Like *nco* lettuce, *chi* mutants of *A*. *thaliana*^30,37^, *P*. *hybrida*^48^, *C*. *chinensis*^49^ and *D*. *caryophyllus*^50^ had low but detectable levels of flavonols. In Arabidopsis, Peer *et al*.^39^ hypothesized that spontaneous isomerisation of naringenin chalcone *in planta* to naringenin, the substrate of the next enzyme in the anthocyanidin biosynthesis pathway, F3H, was responsible for the presence of flavonols.

*KfoA* and *kfoB* lettuce plants are pink-green and accumulate high levels of kaempferol glycosides. Kaempferol has well-documented anti–diabetic, pancreatic β-cell protecting and anti-inflammatory effects^57–59^. It has been reported in lettuce; however, quercetin is the dominant flavonol in most cultivars^28,32–34^. Reported kaempferol levels in lettuce range from 0.0–2.36 mg/100 g fresh weight^28,32–34^, while *kfoA* and *kfoB* lettuce has 103 and 83 mg /100 g fresh weight, respectively, two orders of magnitude higher. Unlike naringenin chalcone, kaempferol is a ubiquitous flavonoid, described from over 400 species (for review, see^57^). However, kaempferol accumulation in *kfo* lettuces is higher than the amounts reported in vegetables considered high in kaempferol, e.g. endive (*Cichorium endiva*), 1.5–9.5^60^; leek (*Allium porrum*), 1.1–5.6^60^ and 11.8^34^; shallot (*Allium fistulosum*), 11.7^34^; potherb mustard (*Brassica juncea*), 48.2^34^; kale (*Brassica oleracea* var. *acephala*), 5.1^34^, 21.1^60^ and 47.0^61^; broccoli (*Brassica oleracea* var. *italica*), 2.1^34^ and 6.0^61^; choi sum cabbage (*Brassica rapa* var. *parachinensis*), 2.0–3.7^32^; turnip tops (*Brassica campestris*), 3.1–6.4^60^; spinach (*Spinacia oleracea*), 4.9–9.0^32^; radish root (*Raphanus sativus*), 0–4.1^34^; toona leaf (*Toona sinensis*), 41.7^34^ and 60.4^33^; Chinese boxthorn shoot (*Lycium barbarum*), 44.6^33^, rocket (*Eruca sativa*) 36.5^33^ and water cress (*Nasturtium officinale*) 35.1^33^ mg kaempferol/100 g fresh weight. The only natural source higher in kaempferol than *kfo* lettuce is caper flower buds (*Capparis* ssp.), which have 85–295 mg kaempferol /100 g fresh weight, providing 8.5–29.5 mg kaempferol per 10 g serving^62^. Therefore, *kfo* lettuces could be valuable dietary sources of kaempferol with one US leaf lettuce serving (85 g) providing ~71–87 mg kaempferol.

*Kfo* phenotypes were caused by mutations in the *F3′H* gene: a mutation of the intron 2 splice acceptor site in *kfoA*, and a nonsense mutation in *kfoB*. In lettuce, no *f3′h* mutant has been described, but in a study of 240 lettuce accessions five genes were identified as *F3′H*, three of which were expressed and two of which (including the *F3′H* gene mutant in *kfoA and kfoB*) carried expressed Qualitative Trait Loci (eQTL) for flavonoid composition^11^. The lettuce *f3′h* phenotype is very similar to *A*. *thaliana f3′h* mutants (called *tt7*), which accumulate kaempferol, and the anthocyanin pelargonidin^40,41^ that differs from cyanidin by the lack of 3′-hydroxylation. *Kfo f3′h* mutant lettuces also accumulate pelargonidin, although pelargonidin levels in *kfo* are much lower than cyanidin levels in red parent line *cv*. Firecracker (Table 1). This difference suggests reduced substrate specificity of the DFR enzyme for its substrate in *f3′h* mutants, dihydrokaempferol, compared to its substrate in wild type lettuce, dihydroquercetin (Fig. 1). Interestingly, *f3′h* mutants in morning glory (*Ipomoea* ssp.) accumulate pelargonidin derivatives producing magenta, pink or fuschia flowers^63^. In carnation (*D*. *caryophillus*), *f3′h* mutants have pink petals, accumulating a pelargonidin glycoside, while plants with functional *F3'H* have purple petals accumulating a cyanidin glycoside^64^.

In plants, many environmental stresses trigger the accumulation of antioxidants including flavonoids and other phenolics^65^. Flavonoids are hypothesized to act as UV absorbers and reduce the levels of damaging reactive oxygen species^66^. In lettuce, exposure to UV or blue light increases flavonoid levels, but reduces yield (e.g.^67–73^). This effect was observed during different months in the field growth season^71^, and in field^67,69,70^ and greenhouse^68^ experiments, where levels of UV exposure were controlled using UV-blocking cover foils, as well as in controlled growth chambers supplemented by UV or blue light emitting LED diodes^72,73^. UV-induced increase in flavonoid and total phenolic content was observed across different green and red cultivars^74^, indicating that it is a universal phenomenon in lettuce. Armas Gutierrez^75^ reported that continuous exposure to cool fluorescent lights resulted in high accumulation of total phenolics, total antioxidants and total anthocyanins. Therefore, in our experiment, we replicated the growth conditions optimal for high phenolic content determined by Armas Gutierrez^75^.

*A*. *thaliana* flavonoid biosynthesis mutants are more sensitive to high UV^29,30,76^ and visible light stress^77^ than wild type plants. As in lettuce, wild type *A*. *thaliana* plants have a decreased rate of biomass accumulation under high UV stress compared to low UV conditions, but the effects are more severe in flavonoid biosynthesis mutants^29,30^. This sensitivity has been attributed to enhanced photoinhibition^77^ and increased lipid and protein peroxidation^76,77^ in mutants lacking flavonoids that absorb UV and scavenge reactive oxygen species. However, the UV sensitivity of the different *A*. *thaliana* mutants is not equal. The kaempferol-accumulating *f3′h* mutant is less UV sensitive than *chs*, *chi* and *f3h* mutants, which accumulate low levels of flavonols^30^. We found that *nco* and *kfo* lettuces grew somewhat slower than wild type *cv*. Firecracker plants under UV-emitting cool fluorescent lights, though we did not observe visible growth retardation under greenhouse conditions (natural light plus supplemental white light). Under cool fluorescent lights, *kfo* (*f3′h*) lettuce grew faster than *nco* (*chi*) but not as fast as wild type *cv*. Firecracker, similarly to *A*. *thaliana f3′h* and *chi* mutants^30^. Potentially, desirable high biomass and flavonoid levels could be obtained by growing *nco* and *kfo* under low UV conditions, and subjecting them to higher levels UV or blue light before harvest. 3-day supplemental UV treatment for 16 h/day has significantly increased total anthocyanin and antioxidant levels in red leaf lettuce, with no effect on the total leaf biomass^73^. Similarly, 6-day pre-harvest exposure to UV resulted in a 4.6x increase in total anthocyanin content and 2.3x increase in total phenolic content in red leaf lettuce field grown under UV-blocking foil^70^, while total biomass of these plants was not significantly different from those not exposed to UV.

In conclusion, we created and characterized flavonoid biosynthetic mutants in lettuce with potential health benefits. The modified flavonoid profile characterized by record high accumulation of kaempferol and naringenin chalcone may transform lettuce into a food with health benefits. However, animal studies and human clinical trials will be needed to confirm the health benefits of the high flavonoid lettuce varieties described here. Innovative mutagenizing and selection strategies producing higher levels of beneficial phytochemicals could be an important strategy for adding value added output traits to common crops.

## Methods

### EMS mutagenesis of *cv*. Firecracker lettuce seeds

Lettuce *cv*. Firecracker (Johnny’s Selected Seeds) seeds were mutagenized with 0.10 or 0.15% EMS, using the protocol in^75^. In short, seeds (M0) were soaked in distilled water containing 0.1% or 0.15% (v/v) EMS and incubated for 12 h at room temperature in a rotary shaker. Thereafter, the EMS solution was decanted, the seeds were washed five times with 50 ml of distilled water and dried. Mutagenized M1 seeds were planted and grown under standard greenhouse conditions at the Rutgers New Jersey Agricultural Experiment Station (NJAES) glass research greenhouse under the following settings: 25 °C/19 °C day/night temperature, 16 h light/8 h dark photoperiod with natural light supplemented with 400 W high pressure sodium lamps. Inflorescences were individually collected from 1,522 mature M1 plants, and the M2 seed was threshed, dried out in the greenhouse and placed in paper coin envelopes. The envelopes were placed in re-sealable plastic storage bags with desiccant and stored at 4 °C.

### Visual screen of the segregating M2 lettuce population

Selfed and segregated M2 seeds were planted in Sun Gro Propagation Mix (Sun Gro Horticulture, Agawam, MA, USA) in plastic trays. At least 12 M2 seeds were planted if there were more than 12 seeds per M2 line. The trays were placed in growth chambers equipped with cool fluorescent lights (Sylvania F96T12/CW/VHO, Osram Sylvania, Danvers, MA, USA), providing a PAR light intensity of 26.3 mol/m^2^d and UV light intensity of 0.7 mol/m^2^d^75^. Growth chambers were programmed for 16 h light/8 h dark photoperiod, 18 °C/15 °C day/night temperature and 60% relative humidity. Under these conditions, wild type *cv*. Firecracker develops a deep red color. At 4–6 weeks, segregating putative mutants exhibiting color variations, and their siblings with wild-type phenotype were identified and transplanted to 10 cm diameter pots filled with Sun Gro Professional Growing Mix (Sun Gro Horticulture, Agawam, MA, USA). At 12–18 weeks, tissue samples were collected from putative mutants for genetic and phytochemical analysis, and the plants were transplanted to 23 cm diameter, 6 l pots (Nursery Supplies, USA) filled with Sun Gro Professional Growing Mix (Sun Gro Horticulture, Agawam, MA, USA), and placed in the Rutgers New Jersey Agricultural Experiment Station (NJAES) research greenhouse under the following conditions: 25 °C/19 °C day/night temperature, 16 h light/8 h dark photoperiod, with natural light supplemented provided by 400 W high pressure sodium lamps. Individual inflorescences were collected from mature M2 plants; the seeds (M3 generation) were dried, threshed, and stored at 4 °C under the same conditions as M2 seeds.

### Growing Firecracker, *kfo* and *nco* plants for phytochemical analysis

Seeds from selfed *cv*. Firecracker, and from homozygous mutant *kfoA*, *kfoB* and *nco* plants were planted in Sun Gro Propagation Mix (Sun Gro Horticulture, Agawam, MA, USA) in plastic trays. The trays were placed in growth chambers under conditions described above. 26 days after planting, seedlings were transplanted to 10 cm-diameter pots filled with Sun Gro Professional Growing Mix (Sun Gro Horticulture, Agawam, MA, USA), and kept in the growth chamber. 130 days (18 weeks) after seeding, a tissue sample was taken from 6 *cv*. Firecracker, 10 *kfoA*, *kfoB* and *nco* plants, weighed, frozen at −80 °C, then lyophilized.

### Extraction and UPLC-MS/MS analysis of flavonoids from putative flavonoid mutants

To determine the flavonoid composition of putative color mutants, extracts were prepared from lyophilized and ground leaf tissue, using the method described in^18^. In short, leaves were kept at −80 °C prior to lyophilization. Freeze-dried leaves were ground to a fine powder with a mortar and pestle. 50 or 100 mg lyophilized leaf powder was placed in a 15 ml plastic tube, protected from light, then, respectively, 1.5 ml or 3 ml solvent (methanol/water/acetic acid; 85:14.5:0.5 v/v), was added. The leaf powder was vortexed with the solvent for 30 sec, sonicated for 5 min, vortexed for another 30 sec, kept for 10 min at room temperature, and centrifuged at 1700 rcf for 5 min. The supernatant was decanted, then the extraction was repeated twice, and the decanted extracts pooled. The decanted solution was centrifuged at 1700 rcf for 8 min and filtered through 0.45 µm polytetrafluoroethylene (PTFE) filters (Fisher Scientific) for UPLC-MS/MS analysis.

Extracts were separated and analyzed by a UPLC-MS/MS system using the protocol described in^78^. Since this protocol results in the co-elution of chlorogenic acid and cyanidin 3-O-malonylglucoside, we used a modified gradient elution to separate these two compounds. For this protocol, the mobile phase consisted of two components: Solvent A (0.5% ACS grade acetic acid in double distilled de-ionized water, pH 3–3.5), and Solvent B (100% acetonitrile). The initial conditions of the gradient were 95% A and 5% B; for 20 minutes the proportion reached linearly 80% A and 20% B. Within the next 3 minutes the proportion was 5% A and 95% B, which was maintained for 4 minutes. Within the following 3 minutes the gradient was adjusted to initial conditions, and 5 additional minutes were included for equilibration before subsequent injections.

### Acid hydrolysis, UPLC-MS/MS analysis and quantification of flavonoid aglycones and total polyphenol content

Lyophilized lettuce leaves from 18-week old *cv*. Firecracker, *kfoA*, *kfoB* and *nco* plants were ground using a mortar and pestle. Fifty mg leaf powder was placed in a plastic tube and subjected to acid hydrolysis, based on the method of Hertog *et al*.^31^. In short, 4 ml solvent (methanol/water; 62.5:37.5 v/v, 2 g/l tert-butylhydroquinone, Sigma Aldrich) was added, then the mix was acidified with 1.0 ml 6 M HCl, vortexed for a few seconds, and placed in a 90 °C water bath for 2 h. Afterwards, 100% methanol was used to make up the volume of the extract to 10 ml. The extract was then sonicated for 5 min, centrifuged at 2500 rpm for 8 min, and filtered through 0.45 μm PTFE filters (Fisher Scientific) for UPLC-MS/MS analysis. Total polyphenol content of the extracts was measured by a modified Folin-Ciocalteu assay^17^ based on^79^ and^80^.

Extracts were separated and analyzed by a UPLC-MS/MS consisting of the Dionex® UltiMate 3000 RSLC ultra-high-pressure liquid chromatography system, consisting of a workstation with ThermoFisher Scientific’s Xcalibur v. 4.0 software package combined with Dionex^®^’s SII LC control software, solvent rack/degasser SRD-3400, pulseless chromatography pump HPG-3400RS, autosampler WPS-3000RS, column compartment TCC-3000RS, and photodiode array detector DAD-3000RS. After passing through the photodiode array detector, the eluent flow was guided to a Q Exactive Plus Orbitrap high-resolution high-mass-accuracy mass spectrometer (MS). Mass detection was full MS scan with low energy collision induced dissociation (CID) from 100 to 1000 m/z in either positive, or negative ionization mode with electrospray (ESI) interface. Sheath gas flow rate was 30 arbitrary units, auxiliary gas flow rate was 7, and sweep gas flow rate was 1. The spray voltage was 3500 volts (−3500 for negative ESI) with a capillary temperature of 275 °C. The mass resolution was 140,000. Column and run conditions were identical to^78^ apart from that the average pump pressure was 3900 psi for the initial conditions.

Putative formulas of flavonoids and other compounds were determined by isotope abundance analysis on the high-resolution mass spectral data with Xcalibur v.4.0 software and reporting the best fitting empirical formula. Database searches were performed using reaxys.com (Elsevier RELX Intellectual Properties SA) and SciFinder (American Chemical Society).

Quantification was based on external standards of commercially available compounds. Naringenin and naringenin chalcone were purchased from Cerilliant, quercetin from Tocris, kaempferol and cyanidin chloride from Sigma Aldrich. Standards were dissolved in anhydrous methanol (naringenin, naringenin chalcone) or ethanol (cyanidin chloride, quercetin, kaempferol). Additionally, pelargonidin was quantified in cyanidin equivalents.

### Nucleic acid isolation, and genotyping *kfo* and *nco* lettuces

Total cellular DNA was isolated from leaves of lettuces grown in growth chambers, using a modified cetyltrimethylammonium bromide (CTAB) method^81^. Total RNA was isolated using the QIAGEN RNeasy Plant Mini Kit (QIAGEN) according to the manufacturer’s instructions. Nucleic acids were quantified using a NanoDrop UV-Vis spectrophotometer (Thermo Fisher Scientific). cDNA synthesis was performed from total RNA using the High-Capacity cDNA Reverse Transcription Kit (Thermo Fisher Scientific), according to the manufacturer’s instructions. Primers (Supplementary Table S2) were designed based on lettuce ESTs or genomic DNA homologs of *A*. *thaliana CHI* (TAIR AT3G55120), *F3′H* (TAIR AT5G07990) and *F3H* (TAIR AT3G51240). PCR-amplification of the full CDS of *F3H* and *CHI* was performed on *nco* cDNA, and of *F3′H* was performed on *kfoA* and *kfoB* genomic DNA, with the following PCR program: 5 min at 94 °C; 34 cycles of 30 sec at 94 °C, 30 sec at 60 °C, 90 sec at 72 °C; 10 min at 72 °C. PCR products were treated with ExoSAP-IT (Affymetrix), and Sanger sequenced. Raw sequence reads were assembled using SeqMan Pro (DNASTAR). Of the M2 generation, one *kfoA* mutant and seven wild type siblings, as well as one *kfoB* and five wild type siblings were genotyped for their *F3′H* alleles. Fifteen *kfoA* mutants and five wild-type siblings were genotyped for their *F3′H* alleles in the M3 generation. One M2 generation *nco* mutant and four wild type siblings were genotyped for their *CHI* alleles. Five *nco* mutants and fourteen wild-type siblings were genotyped for their *CHI* alleles in the M3 generation.

## Supplementary information

Supplementary Information

## Data Availability

The following sequences have been deposited in the NCBI database: *cv*. Firecracker *CHI* (MG981123), *nco chi1* (MG981124), *cv*. Firecracker *F3′H* (MG981125), *kfoA f3′h1* (MG981126), *kfoB f3′h2* (MG981127).
