# Supplementary material for: Genetic and Phytochemical Characterization of Lettuce Flavonoid Biosynthesis Mutants
Source: Sci Rep. 2019 Mar 1;9:3305. doi: 10.1038/s41598-019-39287-y (PMC6397293; doi:10.1038/s41598-019-39287-y)
Supplement: Supplementary file 1 — Supplementary Information [file 41598_2019_39287_MOESM1_ESM.pdf]

Genetic and Phytochemical Characterization of Lettuce Flavonoid Biosynthesis Mutants

Csanad Gurdon*, Alexander Poulev, Isabel Armas, Shukhratdzhon Satorov, Meg Tsai, Ilya Raskin

Affiliation
Department of Plant Biology, Rutgers University, 59 Dudley Road, New Brunswick NJ 08901-8520

Corresponding author e-mail
gurdon@sebs.rutgers.edu

Supplementary Figure S1. Naringenin chalcone hesoxide and malonylhexoside are identified in *nco* lettuce extract based on MS and UV spectra.

Supplementary Figure S2. Chromatograms of non-hydrolyzed lettuce extracts from 18-week old plants.

Supplementary Figure S3. Chromatograms of acid hydrolyzed lettuce extracts from 18-week old plants.

Supplementary Table S1. Phenotype segregation ratios in individual *kfoA*, *kfoB* and *nco* lines.

Supplementary Table S2. Primers for PCR amplification and sequencing of *CHI*, *F3H* and *F3'H* in lettuce.

Supplementary Figure S1. Naringenin chalcone hesoxide and malonylhexoside are identified in *nco* lettuce extract based on MS and UV spectra. Shown are (+) ESI MS Total Ion Current (TIC); m/z 100-1,000 and corresponding UV spectra, and (+) ESI MS and relative absorbance spectra of select peaks. (+) ESI MS TIC of (**a**) naringenin, (**c**) prunin (naringenin-7-O-glucoside), (**e**) naringenin chalcone, (**g**) *nco* methanolic extract; UV absorbance spectra of (**b**) naringenin at 290 nm, (**d**) prunin at 290 nm, (**f**) naringenin chalcone at 365 nm, (**h**) *nco* methanolic extract at 290 nm, (**i**) *nco* methanolic extract at 365 nm; (+) ESI MS of (**j**) naringenin (retention time (RT) 31.26), (**k**) prunin (RT 9.29), (**l**) naringenin chalcone (RT 31.16), (**m**) *nco* methanolic extract (RT 19.12), (**n**) *nco* methanolic extract (RT 22.52); absorbance spectra of (**o**) naringenin (RT 31.16), (**p**) prunin (RT 9.21), (**q**) naringenin chalcone (RT 31.07), (**r**) *nco* methanolic extract (RT 19.12), (**s**) *nco* methanolic extract (RT 22.52). Note that (+) ESI MS spectra of naringenin (**j**) and naringenin chalcone (**l**) share an identical m/z 273.075 [M+H]. This [M+H] value is also observed in prunin (**k**) and two separate peaks (**m,n**) of the *nco* extract. Therefore, naringenin and naringenin chalcone glycosides cannot be distinguished based on (+) ESI MS spectra. However, naringenin (**o**) and naringenin chalcone (**q**) differ in their UV absorbance spectra. Prunin (**p**) has a similar absorbance spectrum to naringenin. Both peaks of the *nco* extract that share the m/z 273.075 (**r,s**) have an absorption spectrum almost identical to naringenin chalcone, and different from naringenin. Based on their UV and mass spectra as well as literature data [27], we identify the RT19.12 peak as naringenin chalcone hexoside, and the RT22.52 peak as naringenin chalcone malonylhexoside.

Naringenin 20 ng

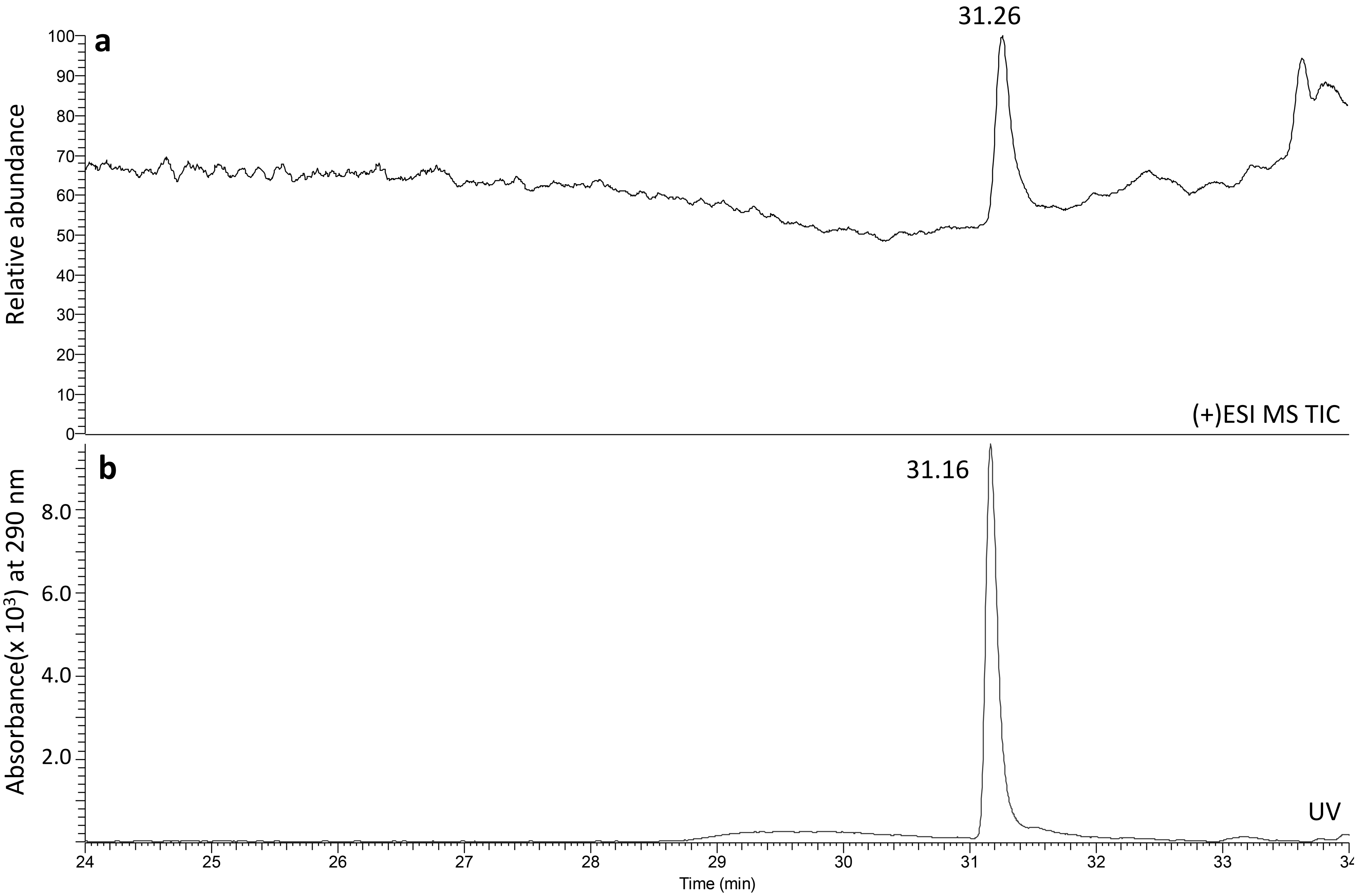

Prunin 100 ng

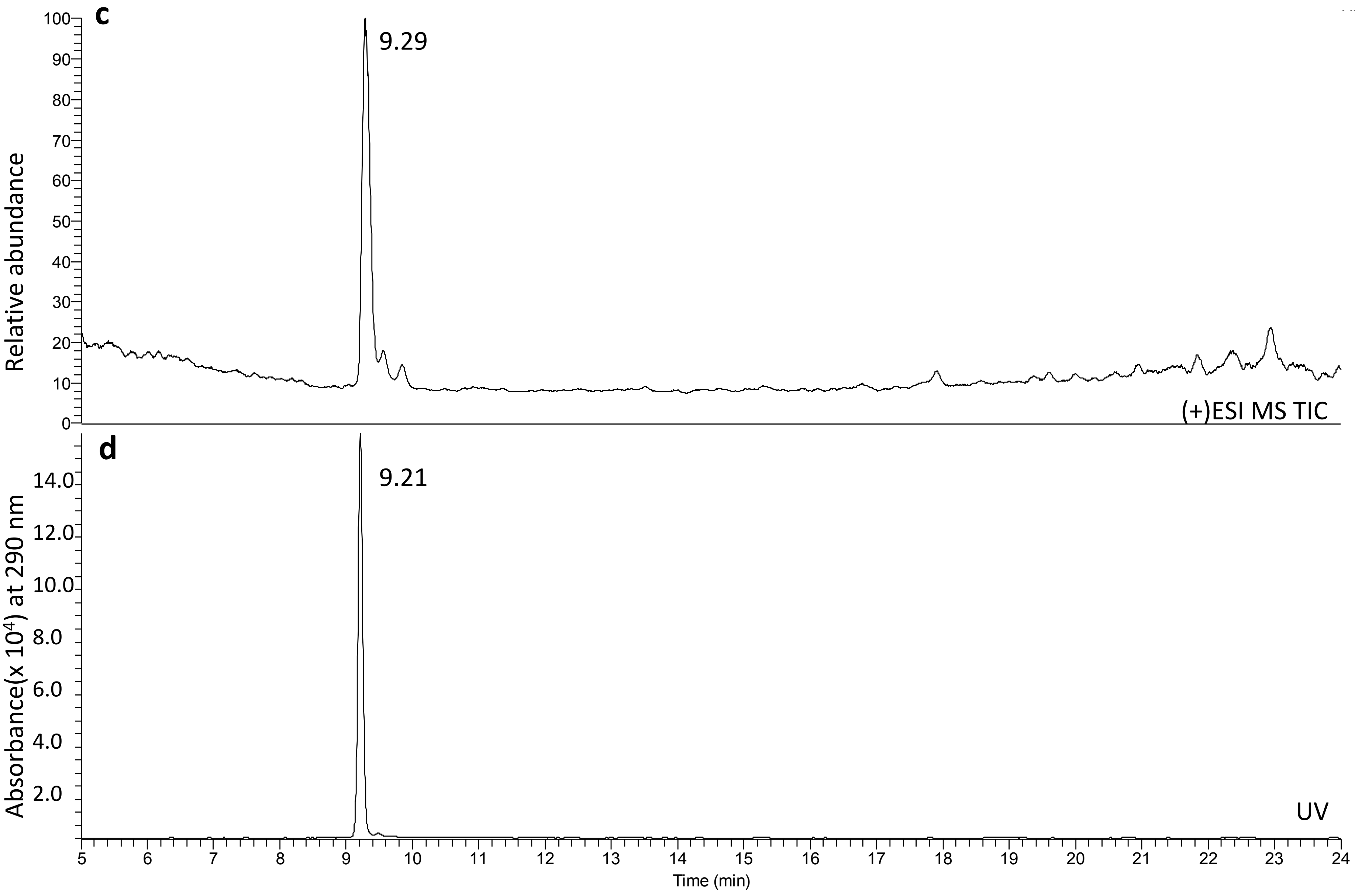

Naringenin chalcone 20 ng

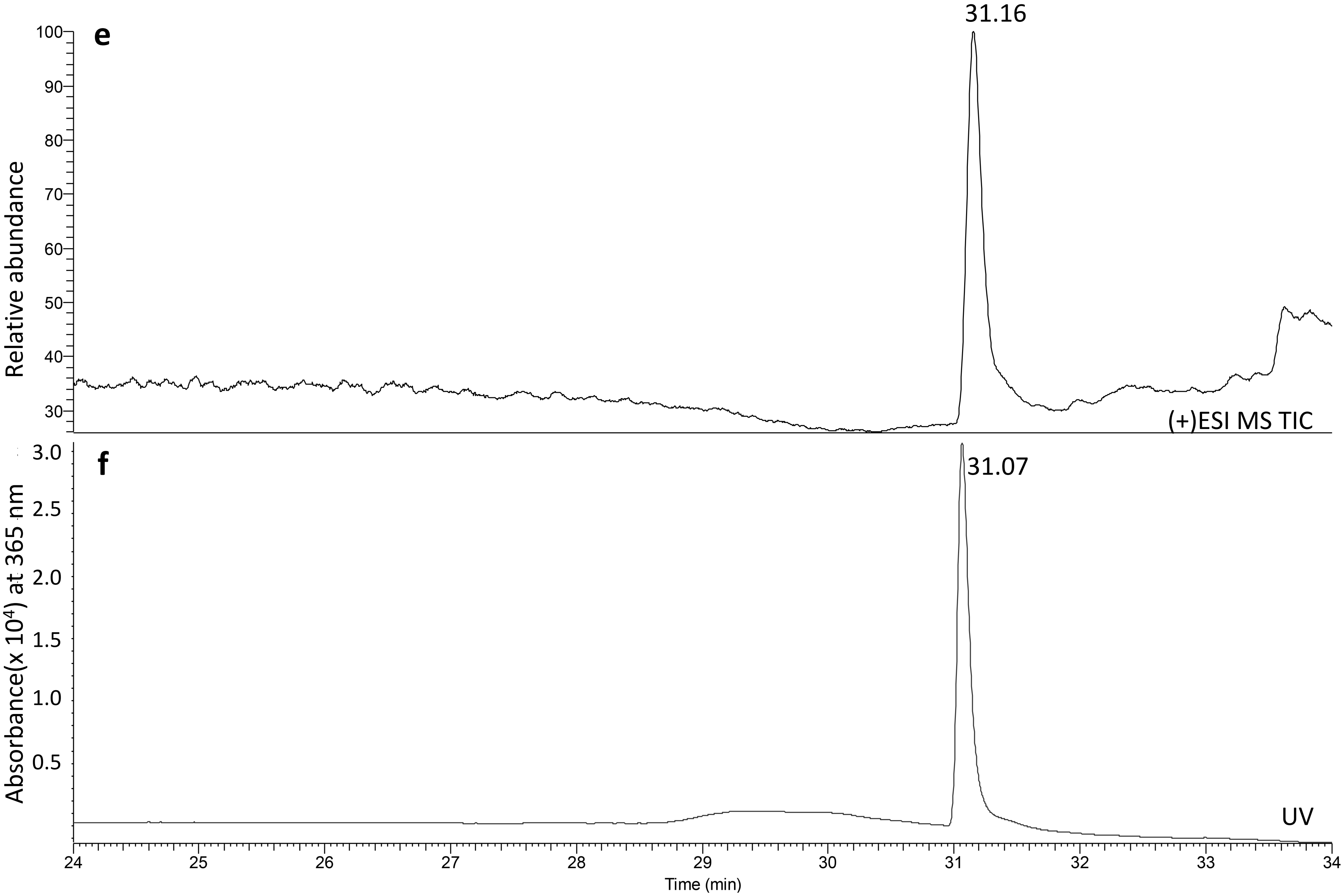

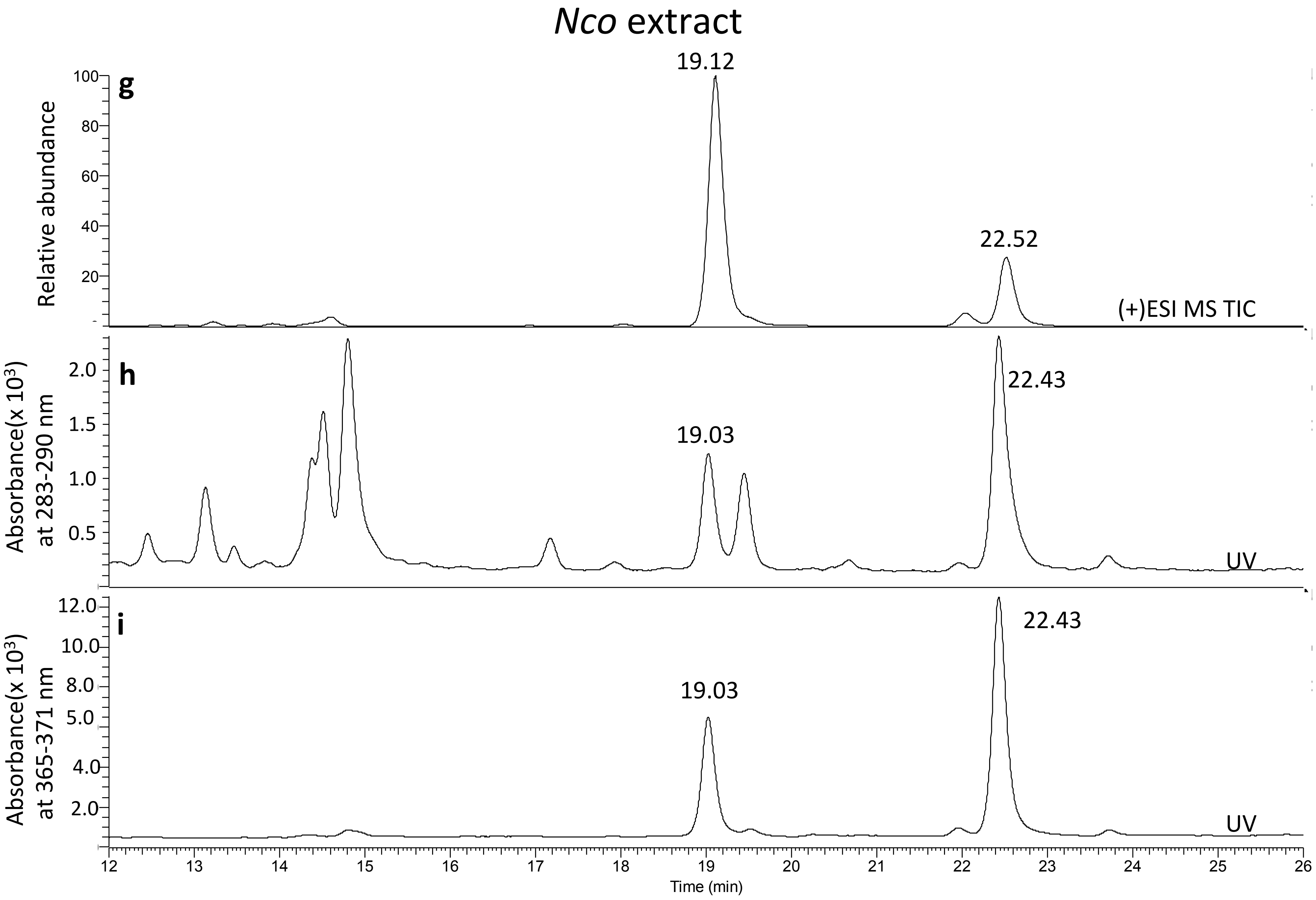

Nco extract
19.12
22.52
g
Relative abundance
(+)ESI MS TIC
h
Absorbance(x 10³) at 283-290 nm
19.03
22.43
UV
i
Absorbance(x 10³) at 365-371 nm
19.03
22.43
UV
Time (min)

Naringenin 20 ng

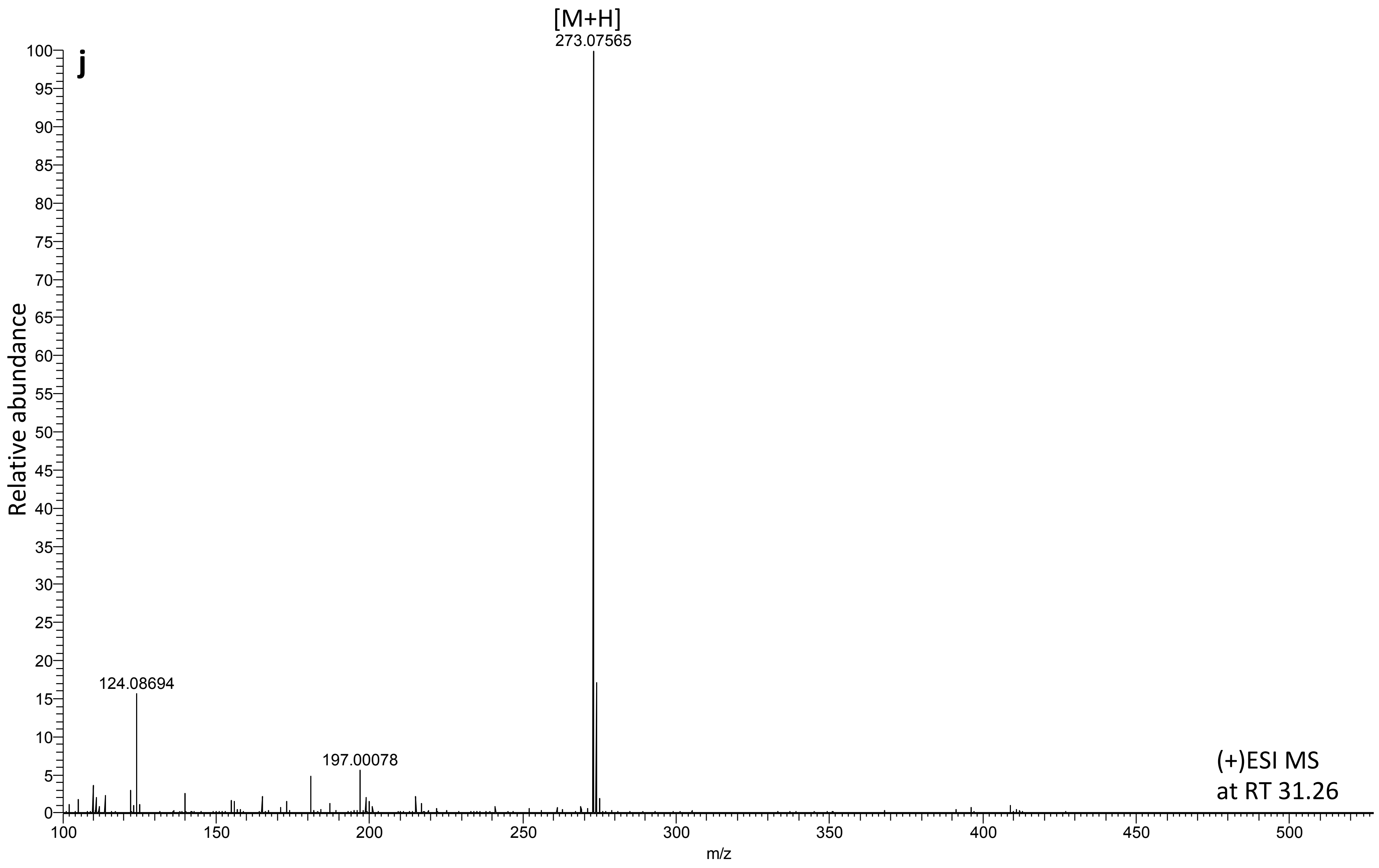

Prunin 100 ng

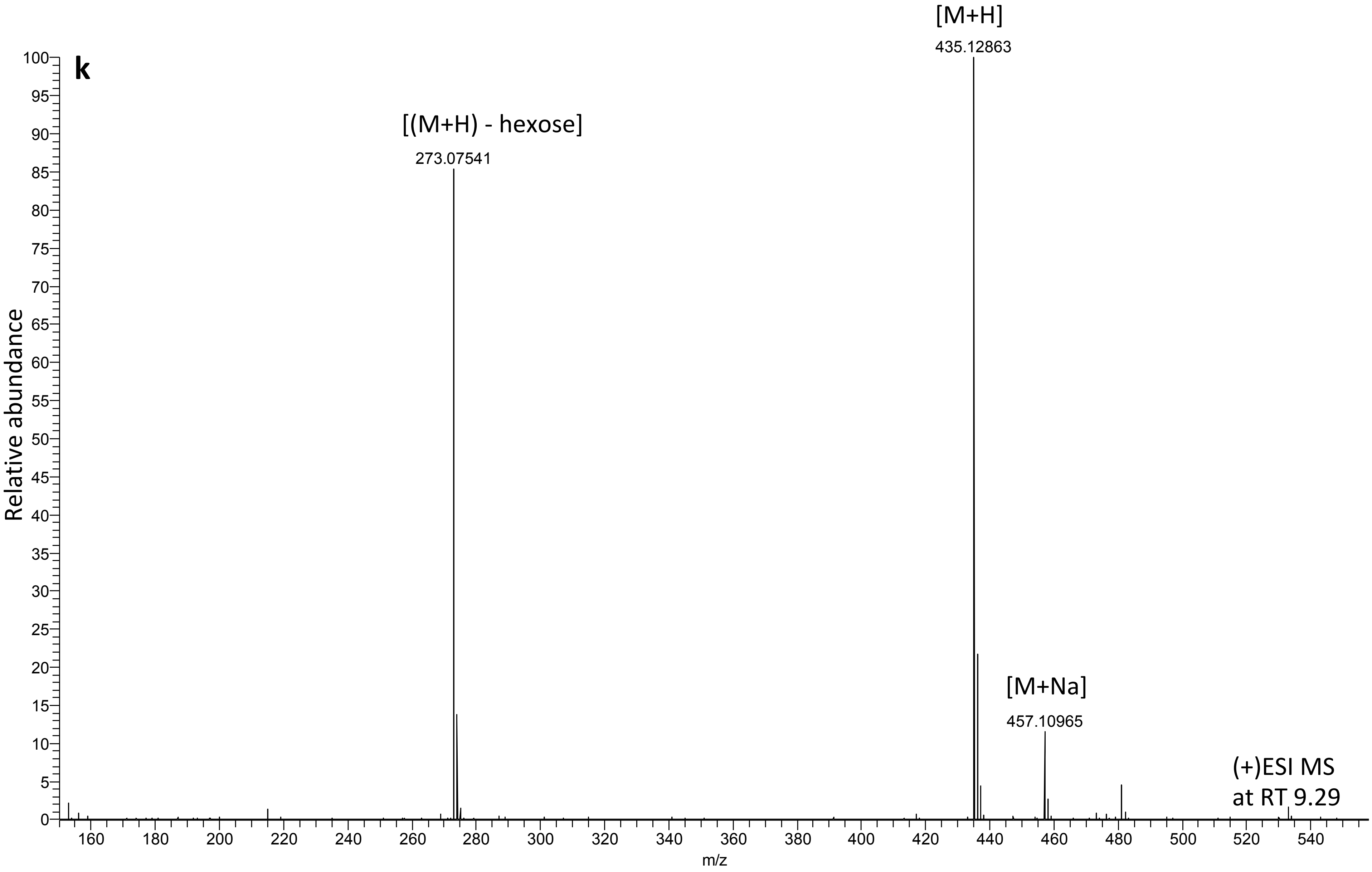

Naringenin chalcone 20 ng

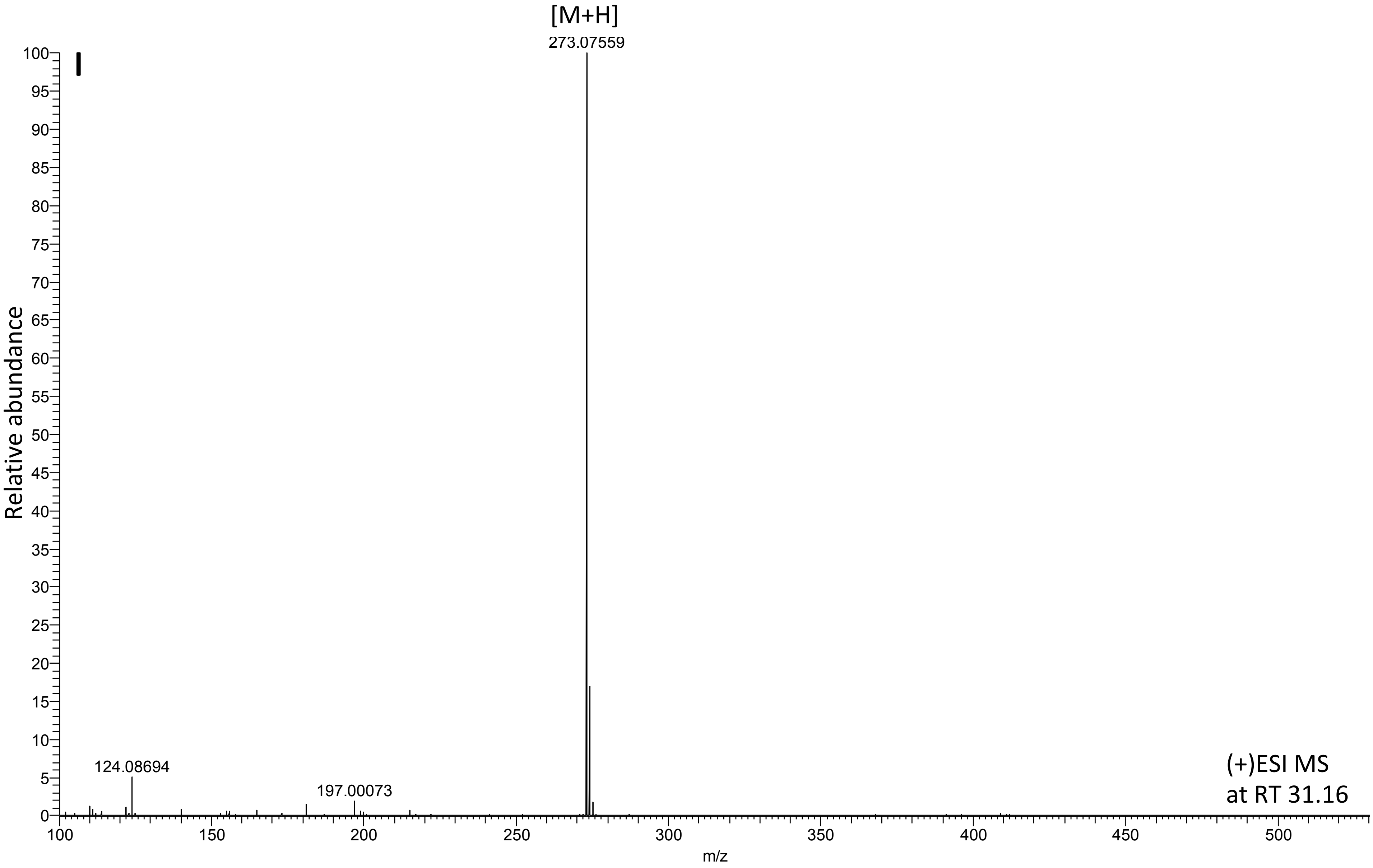

Nco extract

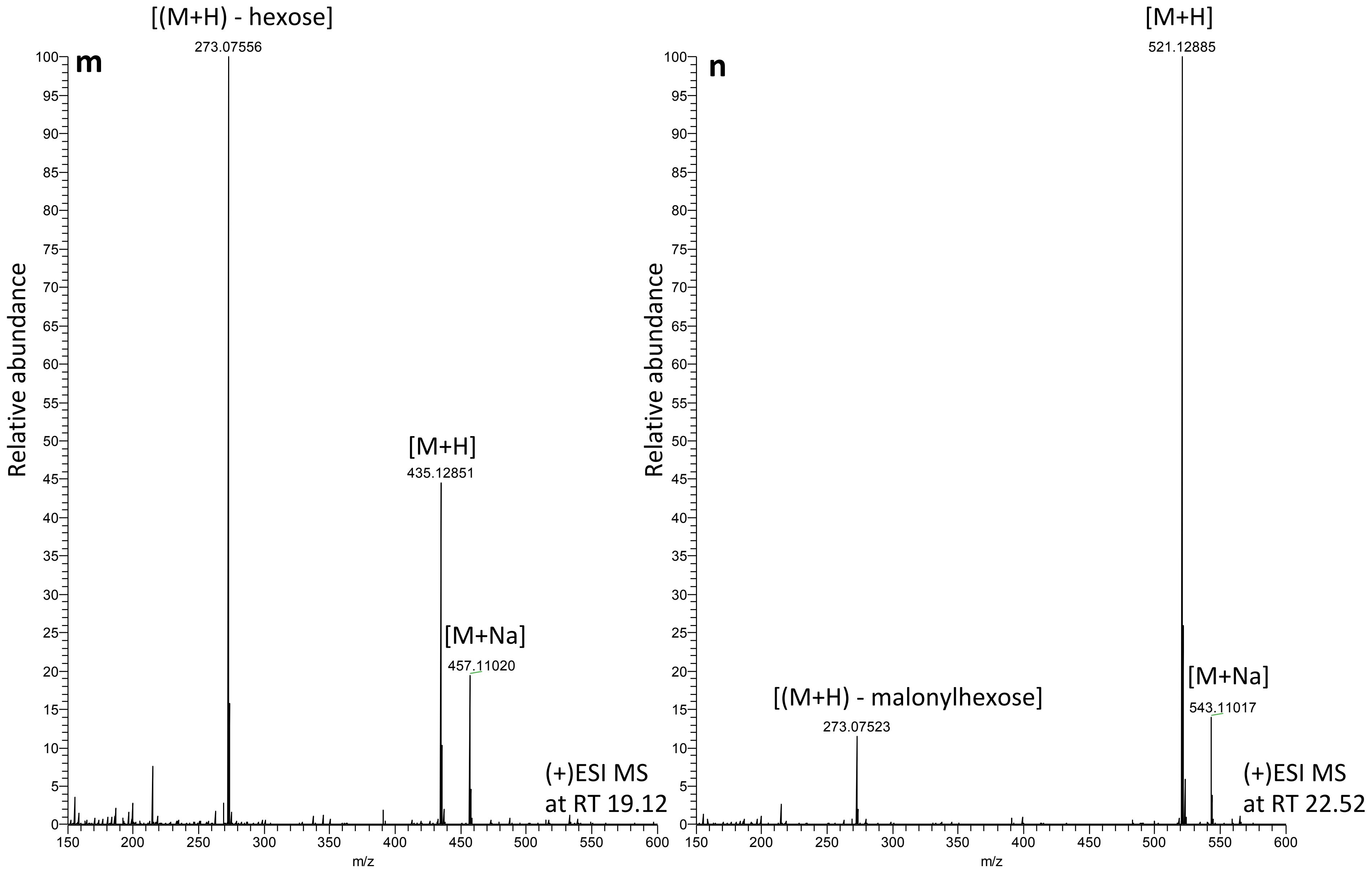

[(M+H) - hexose]
273.07556
m
Relative abundance
[M+H]
435.12851
[M+Na]
457.11020
(+)ESI MS
at RT 19.12
m/z
[M+H]
521.12885
n
Relative abundance
[(M+H) - malonylhexose]
273.07523
[M+Na]
543.11017
(+)ESI MS
at RT 22.52
m/z

Naringenin 20 ng

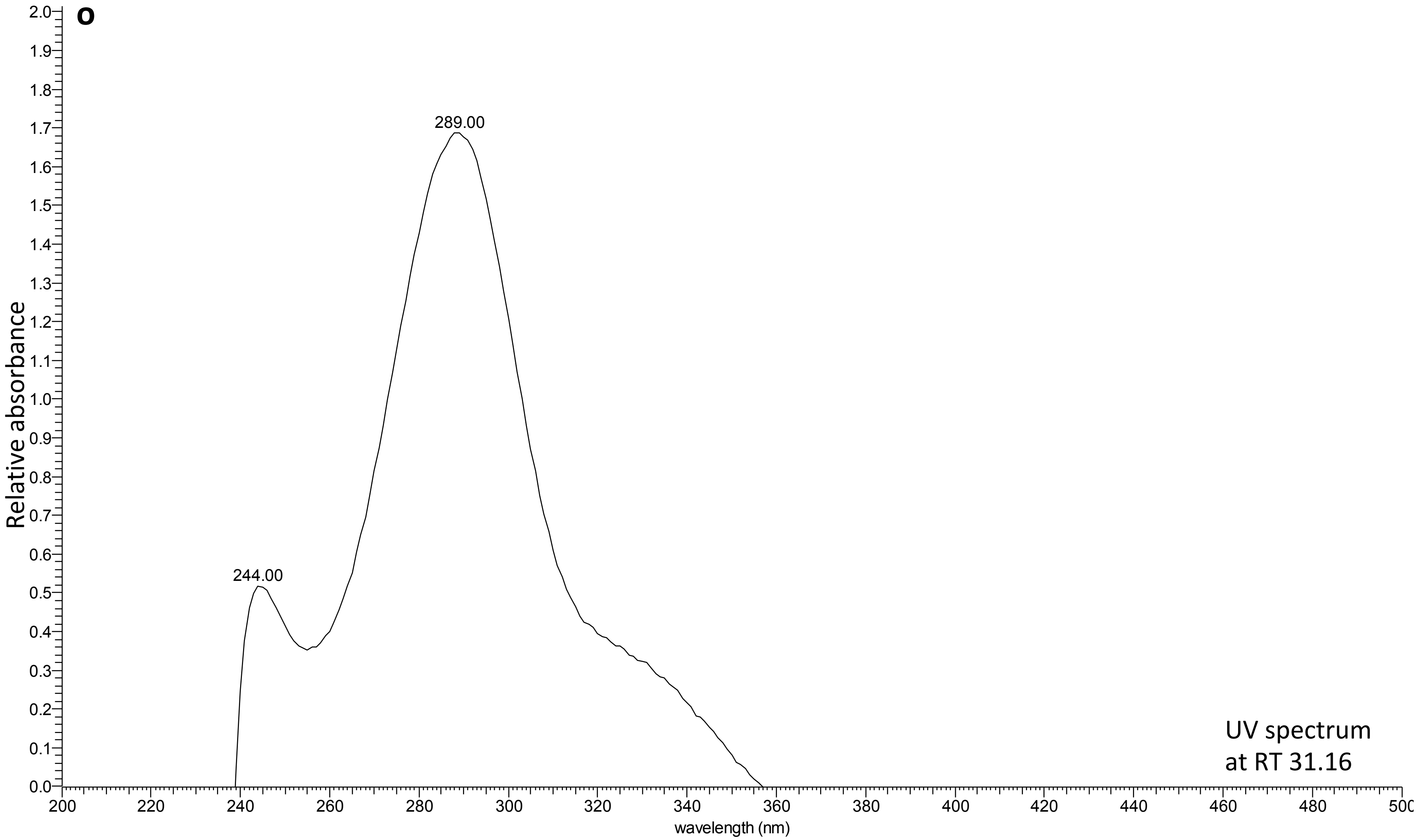

Prunin 100 ng

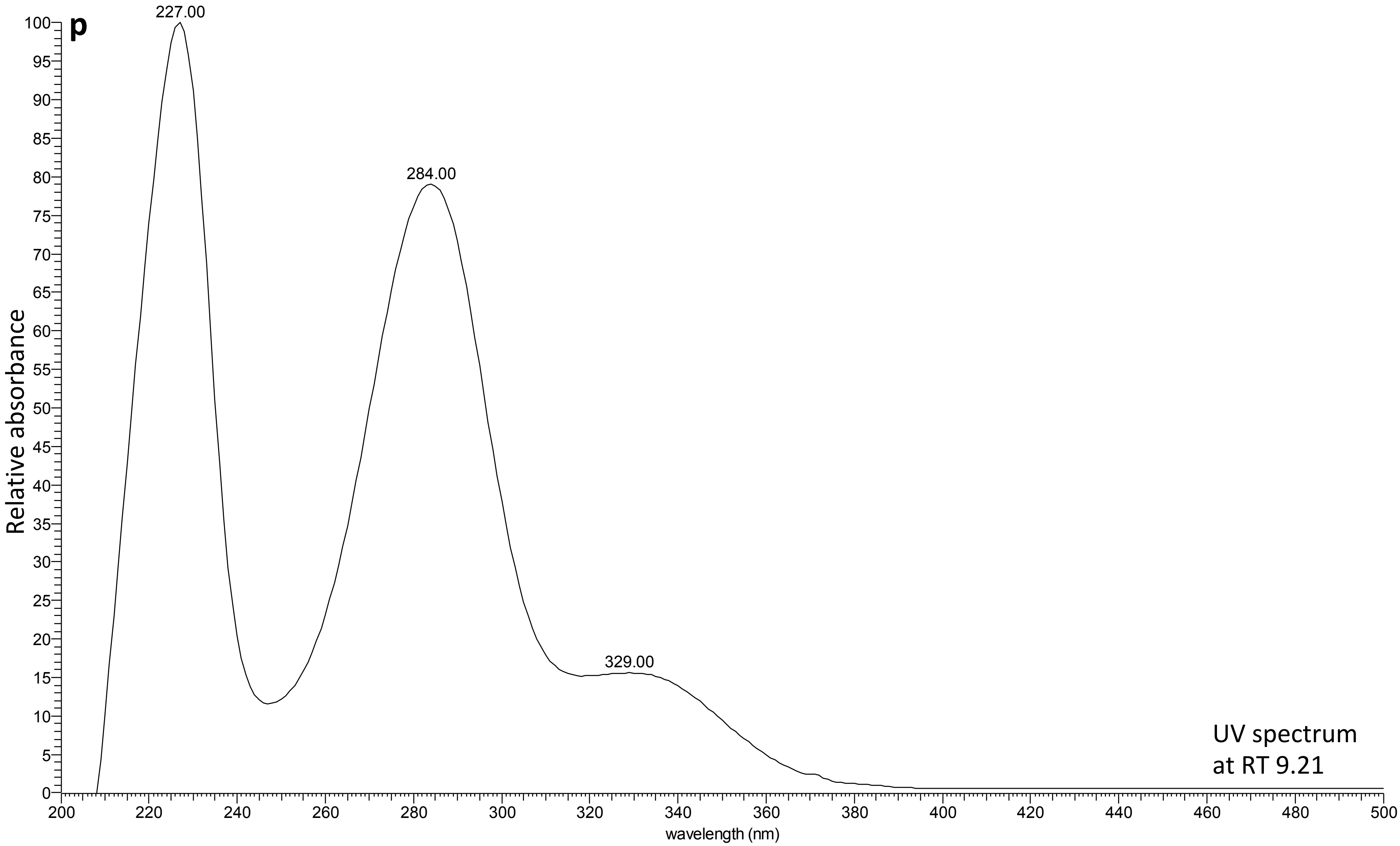

UV spectrum
at RT 9.21

Naringenin chalcone 20 ng

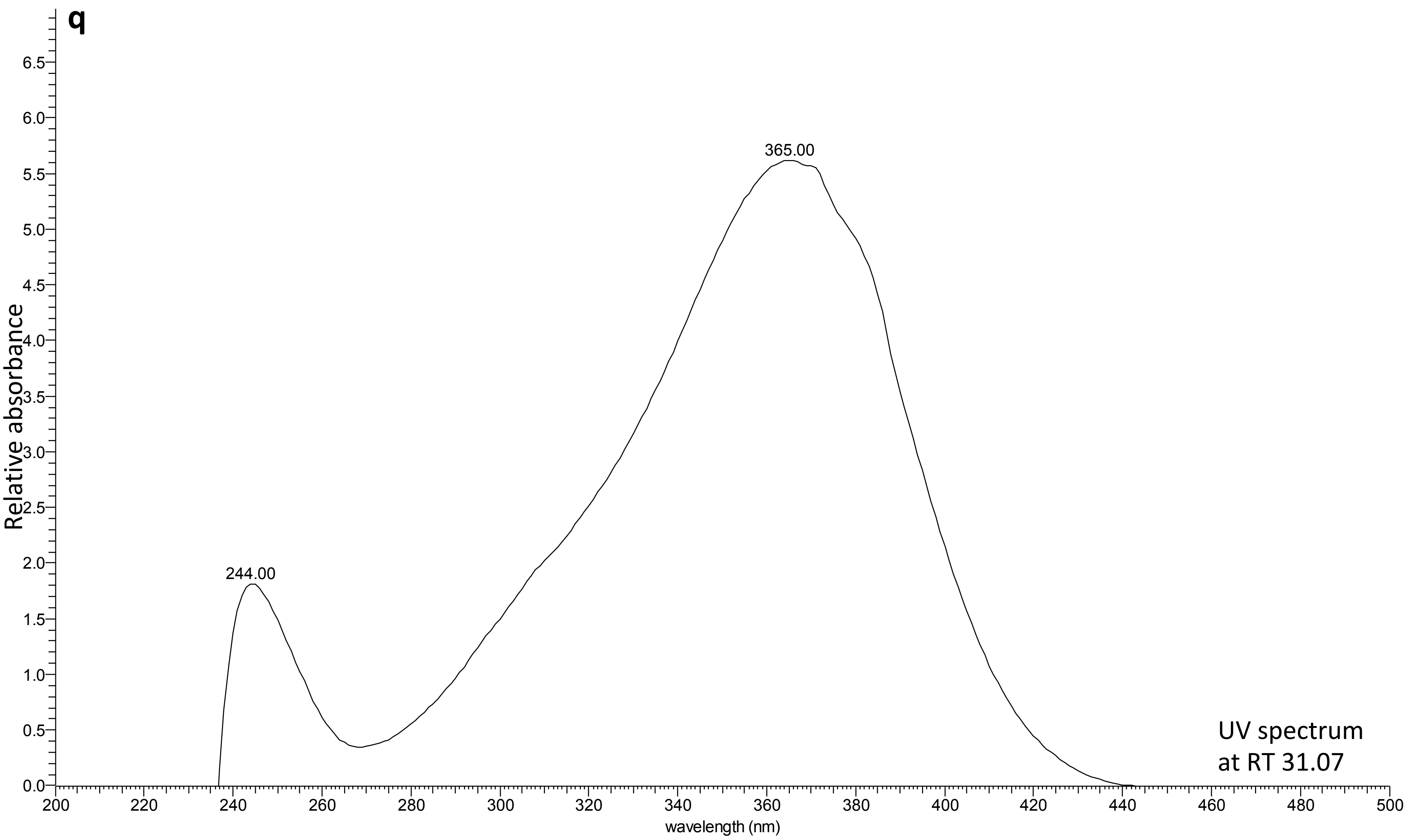

Nco extract

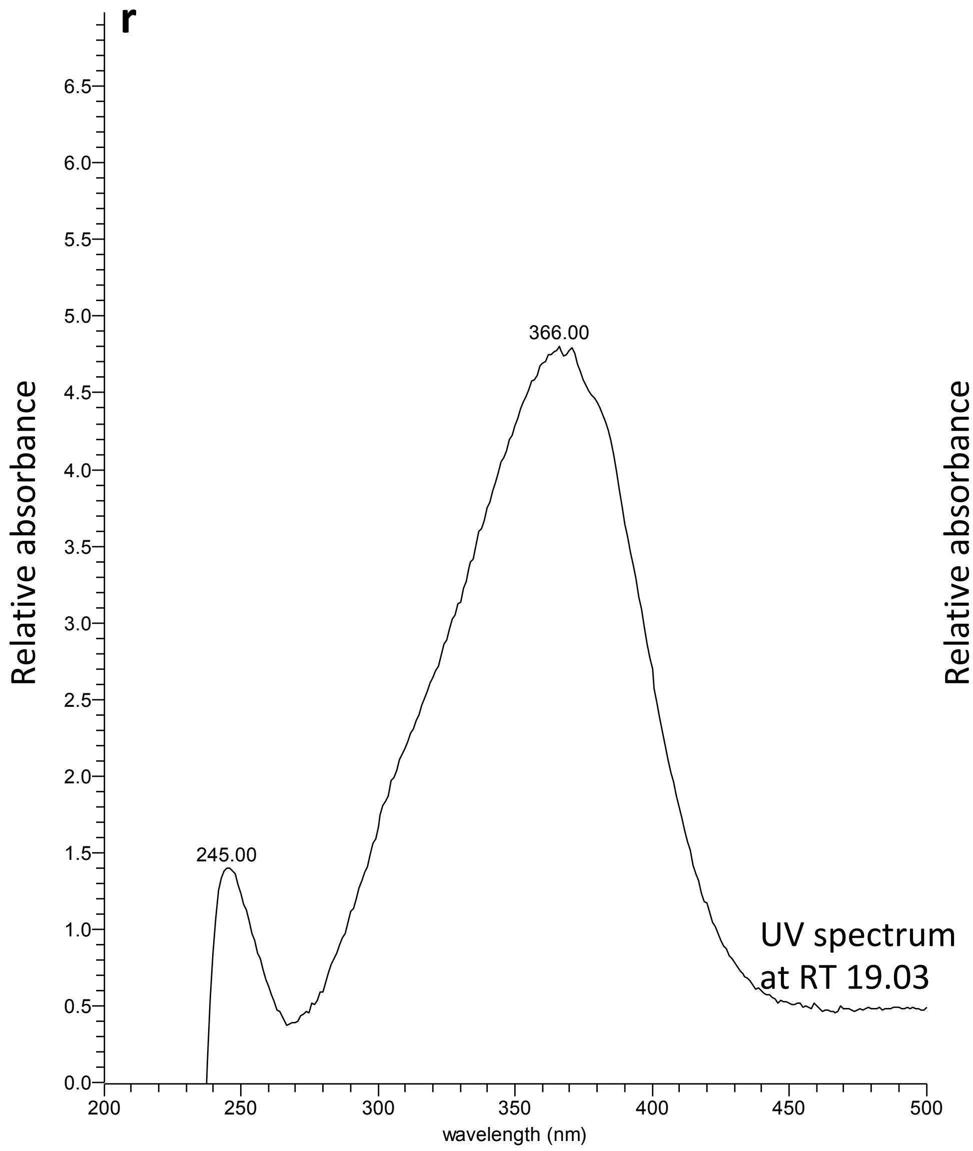
r
Relative absorbance
366.00
245.00
wavelength (nm)
UV spectrum
at RT 19.03

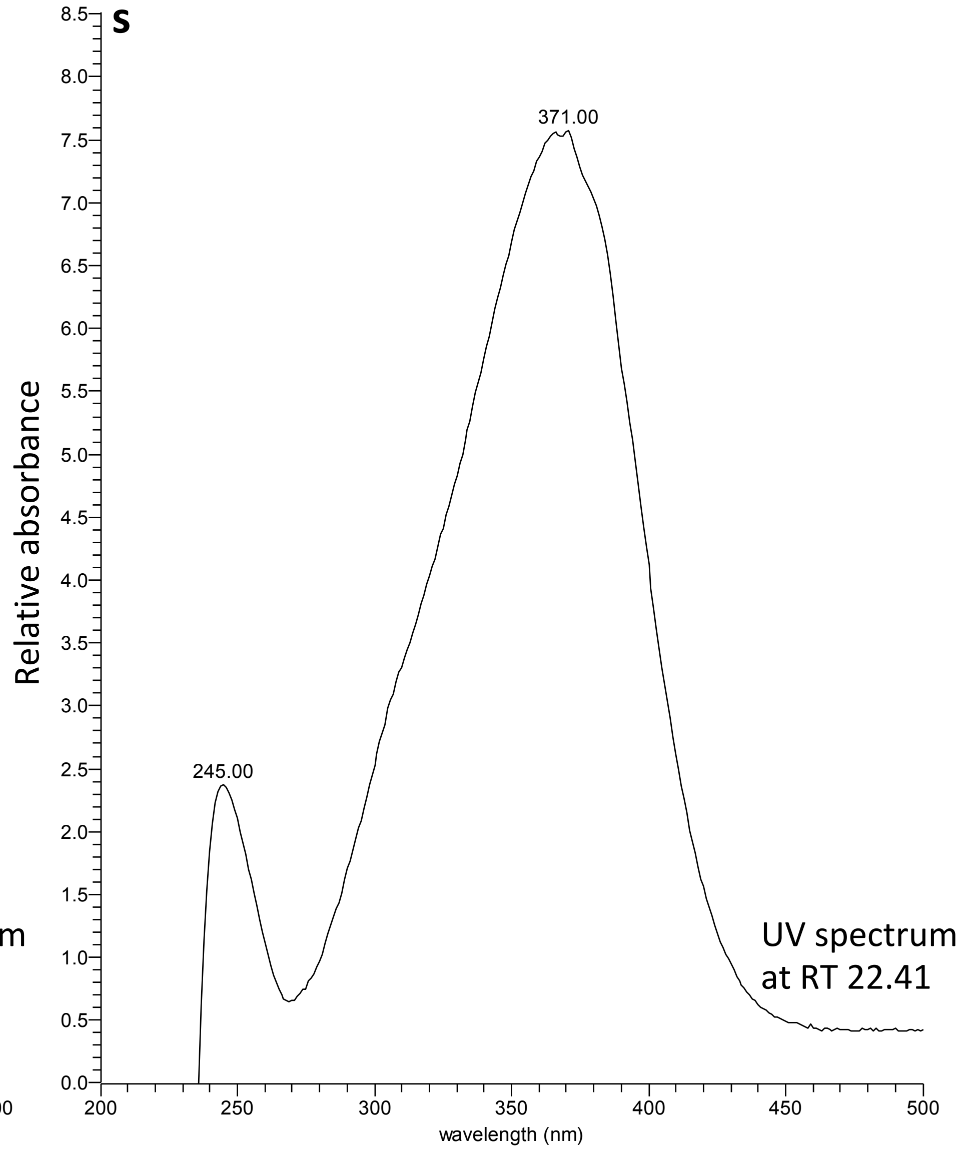
s
Relative absorbance
371.00
245.00
wavelength (nm)
UV spectrum
at RT 22.41

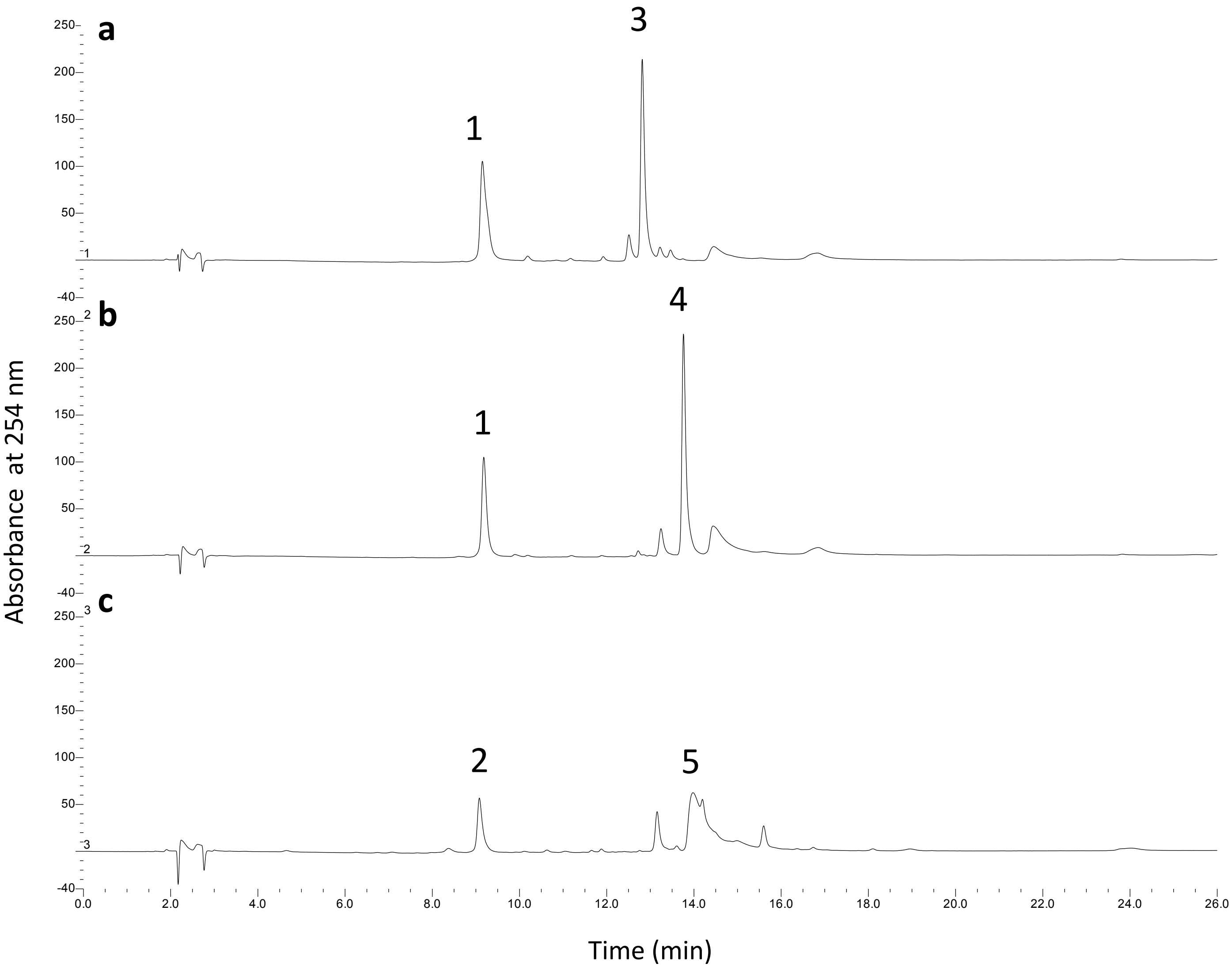

Supplementary Figure S2. Chromatograms of non-hydrolyzed lettuce extracts from 18-week old plants. Shown are representative chromatograms of *cv.* Firecracker (**a**), *kfoA* (**b**) and *nco* (**c**) at 254 nm. Labeled peaks are: co-eluted chlorogenic acid and cyanidin 3-O-malonylglucoside (1), chlorogenic acid (2), quercetin-3-O-malonylglucoside (3), kaempferol 3-O-malonylglucoside (4) mix of naringenin chalcone glycosides (5). Note that not all flavonoid peaks are visible on these chromatograms at the scale shown. An additional run using different solvent gradients was used to separate chlorogenic acid and cyanidin 3-O-malonylglucoside (not shown).

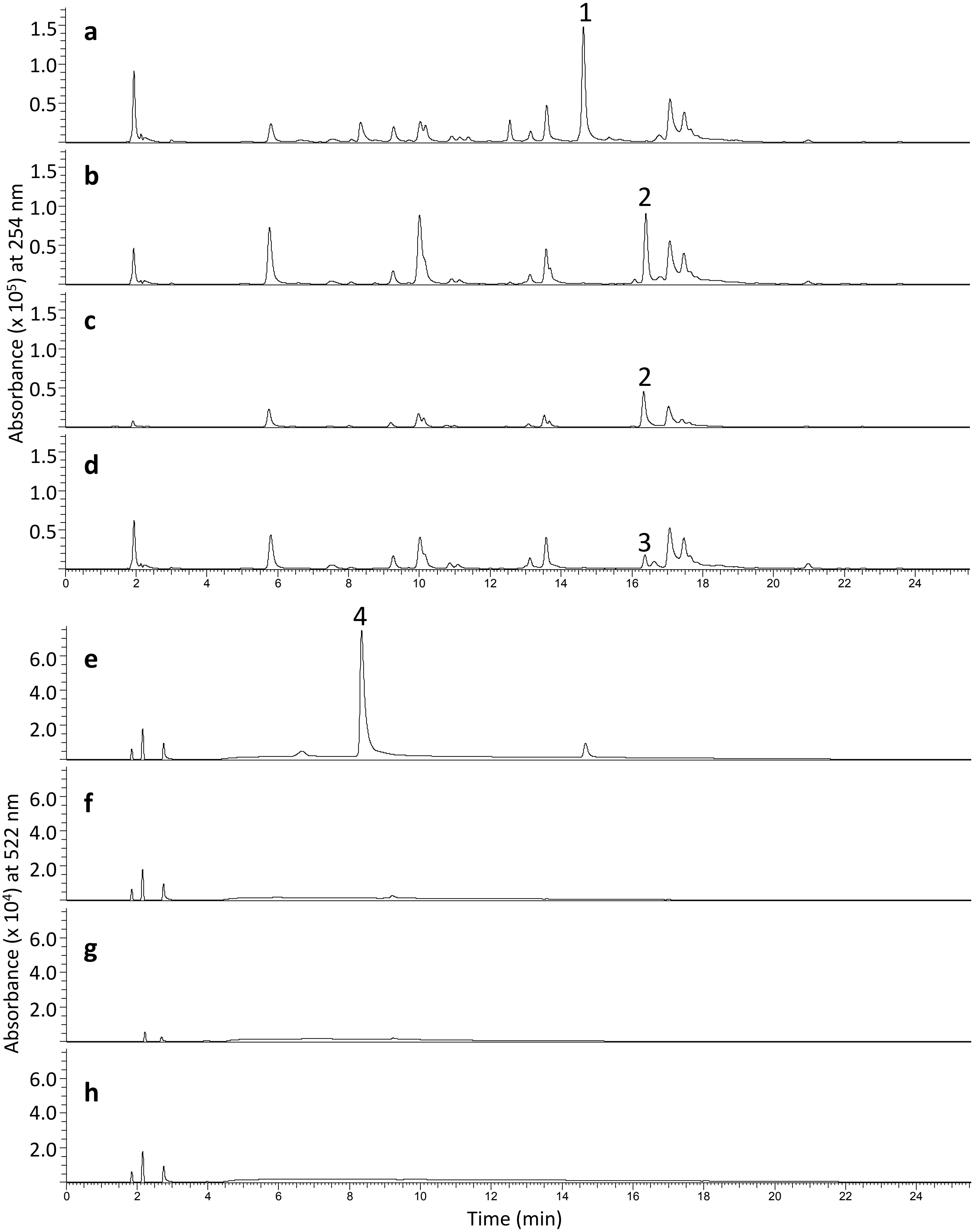

Supplementary Figure S3. Chromatograms of acid hydrolyzed lettuce extracts from 18-week old plants. Shown are representative chromatograms of *cv.* Firecracker (a, e), *kfoA* (b, f) , *kfoB* (c, g) and *nco* (d, h) at 254 nm (a-d) and 522 nm (e-h). Labeled peaks are: quercetin (1), kaempferol (2), naringenin (3), cyanidin (4). Note that not all flavonoid peaks are visible on these chromatograms at the scale shown.

Supplementary Table S1. Phenotype segregation ratios in individual *kfoA, kfoB* and *nco* lines. M, mutant allele; +, wild type allele.

Parent line	Parent genotype	Number of mutant offspring	Number of wild type offspring	Total number of offspring	Percentage of mutant offspring	Expected number of mutant	Expected number of wild type	Chi square value (p=0.05, Chi
kfoA -3-3	M/M	45	0	45	100.0%	45	0	
kfoA- 6-1	M/M	68	0	68	100.0%	68	0	
kfoA -6-101	M/M	92	0	92	100.0%	92	0	
kfoA-6-1-88	M/M	42	0	42	100.00%	42	0	
kfoA-3-3-37	M/M	38	0	38	100.00%	38	0	
kfoA-6-1-25	M/M	45	0	45	100.00%	45	0	
kfoA-6-1-58	M/M	55	0	55	100.00%	4.5	13.5	3.630
kfoA -3	+/M	13	20	33	39.4%	8.25	24.75	3.646
kfoA -6	+/M	11	23	34	32.4%	8.5	25.5	0.980
**Sum *kfoA***	**M/M**	**385**	**0**	**385**	**100.0%**	**385**	**0**	
**Sum *kfoA***	**+/M**	**32**	**53**	**85**	**37.6%**	**21.25**	**63.75**	**7.251**
**Sum *kfoA***	**+/+**	**n/a**	**n/a**	**n/a**	**n/a**			
kfoB -1	M/M	19	0	19	100.0%	19	0	
kfoB-2-2	M/M	15	0	15	100.00%	15	0	
kfoB-2-3	M/M	33	0	33	100.00%	33	0	
kfoB -2	+/M	3	17	20	15.0%	5	15	1.067
kfoB -3	+/M	6	14	20	30.0%	5	15	0.267
kfoB -7	+/M	5	15	20	25.0%	5	15	0.000
kfoB -4	+/+	0	18	18	0.0%	0	18	
kfoB -5	+/+	0	19	19	0.0%	0	19	
kfoB -6	+/+	0	1	1	0.0%	0	1	
**Sum *kfoB***	**M/M**	**67**	**0**	**67**	**100.0%**	**67**	**0**	
**Sum *kfoB***	**+/M**	**14**	**46**	**60**	**19.4%**	**18**	**54**	**2.074**
**Sum *kfoB***	**+/+**	**0**	**38**	**38**	**0.0%**	**0**	**38**	
nco -1	M/M	49	0	49	100.0%	49	0	
nco -1-3	M/M	25	0	25	100.0%	25	0	
nco-2-203	M/M	28	0	28	100.00%	28	0	
nco 1-413	M/M	10	0	10	100.00%	10	0	
nco -2	+/M	12	28	40	30.0%	10	30	0.533
nco -4	+/M	6	18	24	25.0%	6	18	0.000
nco -3	+/+	0	11	11	0.0%	0	11	
nco -6	+/+	0	16	16	0.0%	0	16	
**Sum *nco***	**M/M**	**112**	**0**	**112**	**100.0%**	**112**	**0**	
**Sum *nco***	**+/M**	**18**	**46**	**64**	**28.1%**	**16**	**48**	**0.333**
**Sum *nco***	**+/+**	**0**	**27**	**27**	**0.0%**	**0**	**27**	

Supplementary Table S2. Primers for PCR amplification and sequencing of *CHI, F3H* and *F3'H* in lettuce.

Amplified gene	Primer name	Primer sequence	Product size
CHI	CHI_16F	AAGAGCTAAACCATCAGTCAAACAC	658
	CHI_673R	CCATACCTTCCGATCACCGAC	
	CHI_86F	AGTCTCCAGATCGAATCCGTCG	783
	CHI_868R	TGAGCTTTATTTTAAGATCACATGCGTAG	
F3H	F3H_4F	CACCGTCTTCTTCCTCCCTTG	1186
	F3H_1189R	TCATTACATATGATTGGTGGTAGATGC	
F3'H	F3'H_61F	CCGGTACTCACCATTCAAACGC	1835
	F3'H_1895R	TCCATTGCTATCACTAGAATGTGGG	
	F3'H_155F	TCCCACCGCTCATTTTACCAAC	1815
	F3'H_1969R	TGTTGTTTGATTATTGACGCACAC	
	F3'H_249F	ACTTTTTGCAGTGAGCTCCCATTT	for sequencing
	F3'H_1345R	CAACATCGGTGACTTCATCCCG	
	F3'H_1524F	AAGATGATGCCGATGGAGAGGG	
